# Predictive ability of the American Society of Anaesthesiologists physical status classification system on health-related quality of life of patients after total hip replacement: comparisons across eight EQ-5D-3L value sets

**DOI:** 10.1186/s12891-020-03399-8

**Published:** 2020-07-06

**Authors:** Fitsum Sebsibe Teni, Kristina Burström, Jenny Berg, Reiner Leidl, Ola Rolfson

**Affiliations:** 1 Health Outcomes and Economic Evaluation Research Group, Stockholm Centre for Healthcare Ethics, Department of Learning Informatics, Management and Ethics (LIME), Karolinska Institutet, Stockholm, Sweden; 2 Equity and Health Policy Research Group, Department of Global Public Health, Karolinska Institutet, Stockholm, Sweden; 3 Healthcare Services, Region Stockholm, Stockholm, Sweden; 4 Institute for Health Economics and Health Care Management, Helmholtz Zentrum München, German Research Center for Environmental Health, Neuherberg, Germany; 5 Munich Center of Health Sciences, Ludwig-Maximilians University, Munich, Germany; 6 Department of Orthopaedics, Institute of Clinical Sciences, Sahlgrenska Academy, University of Gothenburg, Gothenburg, Sweden; 7 Swedish Hip Arthroplasty Register, Gothenburg, Sweden

**Keywords:** EQ-5D, Total hip replacement, Value set, American Society of Anaesthesiologists (ASA) physical status classification system, Swedish Hip Arthroplasty Register (SHAR)

## Abstract

**Background:**

American Society of Anaesthesiologists (ASA) physical status classification system and its association with postoperative outcomes has been studied in different diseases. However, there is a paucity of studies on the relationship between ASA class and postoperative health-related quality of life (HRQoL) outcomes following total hip replacement (THR).

The aim of this study was to assess the discriminative abilities of EQ-5D-3L value sets from Sweden, Germany, Denmark and the United Kingdom in relation to ASA classes and these value sets’ abilities to show the predictive performance of ASA classes on HRQoL among THR patients in Sweden.

**Methods:**

A longitudinal study was conducted using data of patients in the Swedish Hip Arthroplasty Register who underwent THR between 2008 and 2016. We included 69,290 pre- and 1-year postoperative records and 21,305 6-year postoperative records. The study examined three experience-based EQ-5D-3L value sets (the Swedish VAS and TTO and the German VAS) and five hypothetical value sets (TTO from Germany and VAS and TTO value sets from Denmark and the UK each). Using linear models, the abilities of the value sets to discriminate among ASA classes and to show the predictive performance of ASA classes on HRQoL score were assessed.

**Results:**

All value sets differentiated among ASA classes and showed the predictive effect of ASA classes on HRQoL. ASA classes were found to predict HRQoL consistently for all value sets investigated, with small variations in prediction error among the models.

**Conclusion:**

ASA classes of patients undergoing THR predicted HRQoL scores significantly and consistently, indicating their importance in tailoring care for patients.

## Background

Total hip replacement (THR) has been described as “the operation of the century” due to its very good long-term outcome and impact on health-related quality of life (HRQoL) [1]. Globally, a million THRs are undertaken annually and are predicted to double around the year 2030 [2]. In Sweden, more than 17,000 THRs were conducted in 2016 amounting to 173 procedures per 100,000 inhabitants [3].

In studying the impact of interventions like THR, different HRQoL instruments are employed to capture patient-reported outcomes (PROs). EQ-5D is among the most commonly used HRQoL instruments [4]. Besides the description of health in five dimensions, mobility, self-care, usual activities, pain/discomfort and anxiety/depression, different value sets are applied to produce a single EQ-5D index. A value set can be described as a formula/algorithm which summarizes the problems reported in the five dimensions of the EQ-5D into a single index by attaching specific weights to each level of severity in all the dimensions. Value sets are developed through studies using various methods (time trade-off, TTO, and visual analogue scale, VAS) and perspectives (experience-based and hypothetical) [4–9] and are typically based on representative national population samples.

There is an increased interest and discussion in applying experience-based perspectives, valuing one’s own health state, in EQ-5D valuation studies [10–19]. Using the VAS method, a number of studies have derived the average population perception for this endpoint as a value set [15, 16, 18, 19]. A respective value set can serve as a comparator for valuations in a patient population, and as a tool to transfer valuations to other populations which is relevant when, for example, results of a register are to be considered in another country. A Swedish value set has used TTO to value one’s own health states, [15], thus combining the experience-basis with the elicitation of preferences which can be used to support the allocation of resources.

The American Society of Anaesthesiologists (ASA) physical status classification system categorizes patients based on operative risk at the time of assessment into six classes. These classes are assigned by anaesthesiologists at preoperative assessments [20]. ASA class I describes a normal healthy patient. In ASA class II, a patient with mild systemic disease is categorized, while a patient having a severe systemic disease is classified under ASA class III. A patient with severe systemic disease considered a constant threat to life is classified into ASA class IV. An individual who is near death and expected not to survive without the surgery is categorized in ASA class V. A patient declared brain dead whose organs are being removed for donor purposes is categorized as ASA class VI [21].

The use of ASA class preoperatively and its association with postoperative outcomes has been studied in different diseases by looking at various outcomes. This was reported by two literature reviews which identified a number of studies in disease areas such as hepatic resection, and surgeries involving gastrointestinal, genitourinary, and cardiac diseases, including hip fracture services, elective total hip, and total knee surgery. Studies with findings suggesting no relationships between ASA class and postoperative outcomes were also identified [22, 23]. A number of studies assessing surgical interventions for hip-related problems showed that preoperative ASA class was related to postoperative complications and readmissions [24]; functional recovery in terms of rehospitalisation and walking ability among others [25]; as well as HRQoL [26]. Similarly, significant differences between the ASA class and postoperative mortality and PRO was reported by a study assessing outcomes of hip and knee arthroplasty [27].

Despite the above findings, there is a paucity of studies assessing the possible relations of ASA class with postoperative HRQoL in the context of elective THR. Users of HRQoL evidence may have different requirements regarding the population doing the valuation and the methods used. The present study aimed at comparing the discriminative abilities of EQ-5D-3L value sets from Sweden, Germany, Denmark and the UK in relation to ASA classes; and comparing the predictive ability of ASA class on HRQoL using these value sets based on the data of patients who underwent THR in Sweden.

## Methods

### Study design

This longitudinal study was conducted based on data of patients who underwent THR in Sweden who were registered in the Swedish Hip Arthroplasty Register (SHAR).

### Sampling

Records of patients who underwent THR in the eight-year period between 2008 and 2016, 128,362 were retrieved from SHAR. Among the records of patients with bilateral operations, only the first ones were included. In addition, records with reoperations occurring between primary operation and time to follow-up were excluded. From the remaining 107,715 records, 69,290 complete records for preoperative and 1-year postoperative follow-ups were included in the study. Among these, 21,305 complete records were included for the 6-year postoperative follow-up. The sampling procedure is illustrated in Additional file 1, Figure S1.

### Data

It has been nearly 40 years since the SHAR’s establishment and it currently holds records of more than 17,000 THRs conducted in the year 2016 alone [3, 28]. Recording of ASA class in SHAR started in 2008 [3]. Data on demographic and clinical variables as well as PROs were retrieved. These included age, sex, height and weight, hip joint diagnosis, laterality as well as ASA class. ASA classes ranged from class I (healthy) to IV (disease considered a constant threat to life) [3]. Body mass index (BMI) of the patients has been categorized according to the WHO classification: < 18.5 (Underweight), 18.5–24.9 (Normal), 25.0–29.9 (Overweight), 30.0–34.9 (Obese class I), 35.0–39.9 (Obese class II) and ≥ 40 (obese class III) [29].

PROs data extracted included the EQ-5D-3L instrument, hip pain levels, and Charnley classes. The EQ-5D-3L instrument includes a five-dimension descriptive system (mobility, self-care, usual activities, pain/discomfort, and anxiety/depression) with three severity levels (no, some and severe problems) and a patient-reported EQ VAS component [3]. Self-reported data on hip pain experienced by patients in the past 4 weeks is provided in five levels: ‘none’, ‘very mild’, ‘mild’, ‘moderate’ and ‘severe’ [3]. The Charnley classification comprises three classes: Patients with one hip involved (Charnley class A), both hips involved in whom no other condition interferes with walking (Charnley class B), and patients with other medical factors, in addition to hip(s), contributing to limited walking ability (Charnley class C) [30].

### Value sets compared

A total of eight EQ-5D-3L value sets, from four countries, were employed for comparisons with one another based on the SHAR data on THRs. Patient-reported EQ VAS was also compared with the value sets. Either TTO or VAS valuation was used in the development of the value sets from the four countries. Experience-based perspective was used in the three value sets (the Swedish VAS, TTO and the German VAS) and hypothetical perspective in the other five value sets.

#### The Swedish value sets

These were the Swedish experience-based EQ-5D-3L VAS and TTO value sets. These value sets were developed based on a general population study involving more than 45,000 participants in Sweden (data from 2004 and 2006) using an experience-based perspective. The value sets were estimated through ordinary least square (OLS) regression. The index resulting from the TTO value set ranges from 0.34 to 0.97 for health states 33333 to 11111 while the results of the VAS value set ranges from 17.24 to 88.86 [15].

#### The German value sets

One of the value sets in the comparisons made in the present study was the German experience-based EQ-5D-3L VAS value set [16]. In its development, data on experienced health states were collected from nearly 2000 participants in each of two general population surveys in 2006 and 2007. The estimation of the value set involved generalized linear models and the range of the resulting index is from 0.184 to 0.893 [16]. Another German value set compared in the study was a hypothetical TTO value set prepared based on 36 health states elicited in a study carried out in 1997/98 involving 339 participants. The range of the EQ-5D index was from − 0.20 to 1.0 calculated using additive linear model, i.e. OLS [31].

#### The Danish value sets

Another value set in the comparison was a hypothetical EQ-5D-3L VAS value set from Denmark. The index from this value set ranges from − 0.167 to 1.0. It is based on a study of 1179 participants in 1999/2000 with 16 health states valued by each participant. A random-effects model was used in producing the EQ-5D indices across the health states valued [32]. The Danish hypothetical TTO which was based on a study in 2000 among 1332 participants based on 46 health states was also part of the current study. The final model used in estimating the value sets was a random-effects model. This value set ranges from a worst health state score of − 0.62 to full health with a score of 1, and dead set at 0 [33].

#### The UK value sets

The value sets from the UK, VAS and TTO, were developed using a hypothetical perspective based on data collected in 1993 from 3395 participants. In the data collection, 42 health states were valued and generalized least square regression with additive functional form was used in modelling both value sets. The EQ-5D indices resulting from the VAS value set range from − 0.073 to 1.0 while those of the TTO value set spans an interval of − 0.594 to 1.0 [32, 34].

### Data entry, analysis, and interpretation

#### Baseline characteristics of patients across ASA classes

The data extracted from SHAR was assessed for completeness and checked for erroneous and/or inconsistent entries. The uniformity of distribution of baseline characteristics of patients in SHAR across ASA classes was checked using Cochran-Armitage (for categorical the variables sex, diagnosis and side of operation) and Kendall rank correlation (for the ordinal variables age group and BMI category) [35]. In addition, the strengths of associations were determined through effect size (Cramer’s V) for Cochran-Armitage test. Cramer’s V is categorized as small, medium and large based on the respective degrees of freedom in the chi-square test denoting the magnitude of the association found [36]. Kendall tau coefficient was used to determine the strength of association in Kendall rank correlation test.

#### One-way analysis of variance (ANOVA)

The data from the EQ-5D-3L descriptive system across preoperative, 1-year and 6-year postoperative follow-ups were converted to EQ-5D indices based on all the value sets. One-way analysis of variance (ANOVA), for each time point, was performed to answer the questions whether the value sets were able to discriminate among the different ASA classes. In the one-way ANOVA test, EQ-5D indices from each value set were assessed for the presence of statistically significant differences in mean value across ASA classes. As the assumption of homogeneity of variance was violated, the robustness of the one-way ANOVA test was confirmed by additionally conducting the non-parametric Kruskal Wallis test [37]. The strengths of the associations found were assessed through the effect size measure, Eta squared [38]. The effect sizes 0.01, 0.06 and 0.14 are considered cut-off points for small, medium and large effect sizes respectively [39]. The values denote the proportion of variation in mean HRQoL score explained by the membership of the groups (ASA classes in the present study).

#### Ordinary least squares regression (OLS) and prediction error measures

Ordinary least squares (OLS) regression was performed to assess whether the value sets can show the predictive ability of the ASA class on HRQoL. Dummy variables were created for the ASA classes. In this analysis, patient-reported EQ VAS and EQ-5D indices in a 0 to 100 scale were divided by 100 to facilitate comparison with the other value sets. In the OLS models, ASA classes were assessed as predictors of patient-reported EQ VAS score and the EQ-5D indices from each value set. The models were compared using model estimates, R^2^/ adjusted R^2^ values, mean absolute error (MAE), as well as root mean square error (RMSE).

The two prediction error measures are also presented in normalized MAE and normalized RMSE versions. MAE provides the mean of the absolute values of differences/errors between the observed and predicted values [40]. RMSE measures the error of prediction by first summing up squares of differences between the observed and predicted values and dividing them by the number of cases. The square root of the mean of the squared errors then gives the RMSE [40]. Normalized MAE and RMSE are useful in facilitating comparisons among models with different ranges of values involved, for example, the UK TTO has a range of 1.59 while the Swedish TTO has 0.63. Normalized RMSE has been used in comparing different models in previous studies [41–43].

The above unadjusted models with only ASA classes were adjusted for different independent variables including demographic, clinical and PROs. Assumptions of normality of residuals, constant variance of the error term and absence of significant outliers were assessed through the Breusch-Pagan test of heteroskedasticity, the Q-Q plot of residuals and the Kolmogorov-Smirnov test. Robust standard error (RSE), also known as heteroskedasticity-consistent standard errors were reported instead of standard errors as heteroskedasticity was observed. RSE is a standard error calculated in an approach where homoscedasticity is not assumed [44]. The results of the statistical tests were interpreted using a p-value cut-off at 0.05. All analyses were carried out using the software for statistical computing, R version 3.4.4 [45]. Specific R packages used in the analysis are listed in Additional file 2.

## Results

### Demographic and clinical characteristics of THR patients at baseline

The patients in the study had a mean age of 68.2 years at baseline with nearly two-thirds between 60 and 80 years and more than half (56.8%) being women. Primary osteoarthritis accounted for almost all (92.6%) of the diagnoses leading to THR. All demographic and clinical variables at baseline except sex had statistically significant associations with ASA class, however, these were mostly of small effect size based on Cramer’s V and of lower Kendall tau values (Table 1).

**Table 1:** Distribution of demographic and clinical characteristics of THR patients by ASA class preoperatively (*n* = 69,290)

Variable	ASA class										*P*-value	Cramer’s V / Kendall tau
	Total		Class I		Class II		Class III		Class IV			
	%	n	%	n	%	n	%	n	%	n		
Sex											0.352	0.053
Men	43.2	29,965	46.0	8191	41.2	17,074	47.0	4584	53.0	116		
Women	56.8	39,325	54.0	9631	58.8	24,418	53.0	5173	47.0	103		
Age (years) [mean (SD)]	68.2 (10.2)		62.7 (10.4)		69.4 (9.4)		72.9 (9.2)		73.5 (10.1)		< 0.001	–
Age group (years)											< 0.001	0.282^**d**^
< 50	4.7	3246	11.2	1995	2.6	1094	1.5	151	2.7	6		
50–59	13.5	9351	23.9	4251	10.8	4485	6.1	600	6.8	15		
60–69	34.4	23,822	38.6	6886	34.8	14,453	24.9	2433	22.8	50		
70–79	34.5	23,906	22.4	3986	37.9	15,720	42.2	4122	35.6	78		
80–95	12.9	8965	4.0	704	13.8	5740	25.1	2451	32.0	70		
BMI category^a^											< 0.001	0.155^**d**^
Underweight	0.7	485	0.6	108	0.7	280	0.9	91	2.7	6		
Normal weight	31.0	21,498	39.6	7066	28.9	12,008	24.3	2371	24.2	53		
Overweight	44.3	30,694	46.2	8230	45.2	18,761	37.1	3621	37.4	82		
Obese class I	18.5	12,806	12.0	2144	20.0	8291	23.8	2323	21.9	48		
Obese class II	4.6	3168	1.3	231	4.5	1859	10.8	1056	10.0	22		
Obese class III	0.9	639	0.2	43	0.7	293	3.0	295	3.7	8		
Diagnosis											0.001	0.037
Primary OA^b^	92.6	64,142	92.2	16,426	93.2	38,687	90.6	8838	87.2	191		
Other^c^	7.4	5148	7.8	1396	6.8	2805	9.4	919	12.8	28		
Side of operation											0.001	0.016
Right	55.7	38,623	54.4	9692	56.2	23,330	56.2	5482	54.3	119		
Left	44.3	30,667	45.6	8130	43.8	18,162	43.8	4275	45.7	100		

^a^ BMI categories: Underweight: < 18.5; Normal: 18.5–24.9; Overweight: 25.0–29.9; Obese class I: 30.0–34.9; Obese class II: 35.0–39.9; Obese class III: ≥ 40

^b^ Osteoarthritis

^c^ Other – Other secondary osteoarthritis, femoral head necrosis, sequelae after childhood disease in the hip joint, inflammatory joint disease, other

^d^ Kendall tau coefficient

### Reported problems in the EQ-5D-3L dimensions by ASA class

The numbers of health states recorded pre- and 1 year postoperatively were 159 and 174 respectively. Preoperatively, nearly all patients reported problems in the mobility dimension across ASA classes. The highest proportion of severe problems were reported in the pain/discomfort dimension in all ASA classes. The proportions of problems decreased 1 year postoperatively across all the dimensions. Severe problems were reported more frequently among higher ASA classes in all the dimensions (Additional file 2, Table S2).

### Patient-reported EQ VAS score

The mean patient-reported EQ VAS score (56.0 [SD = 22.3]) increased to 76.7 (SD = 19.8) 1 year postoperatively. The mean and median patient-reported EQ VAS scores were lower in higher ASA classes, in more severe Charnley classes and at worse hip pain levels both pre- and 1 year postoperatively (Additional file 1, Figure S2-S5). Histograms showing distributions of the EQ-5D indices pre- and 1 year postoperatively are provided in Additional file 1, Figures S6 and S7.

### Mean EQ-5D index

The mean EQ-5D indices based on all the value sets increased from pre- to 1-year postoperative follow-up. The Swedish TTO showed the highest index at both follow-ups while the UK TTO recorded the lowest (Additional file 2, Tables S2).

### Discriminative abilities of value sets among ASA classes

**Fig. 1:**
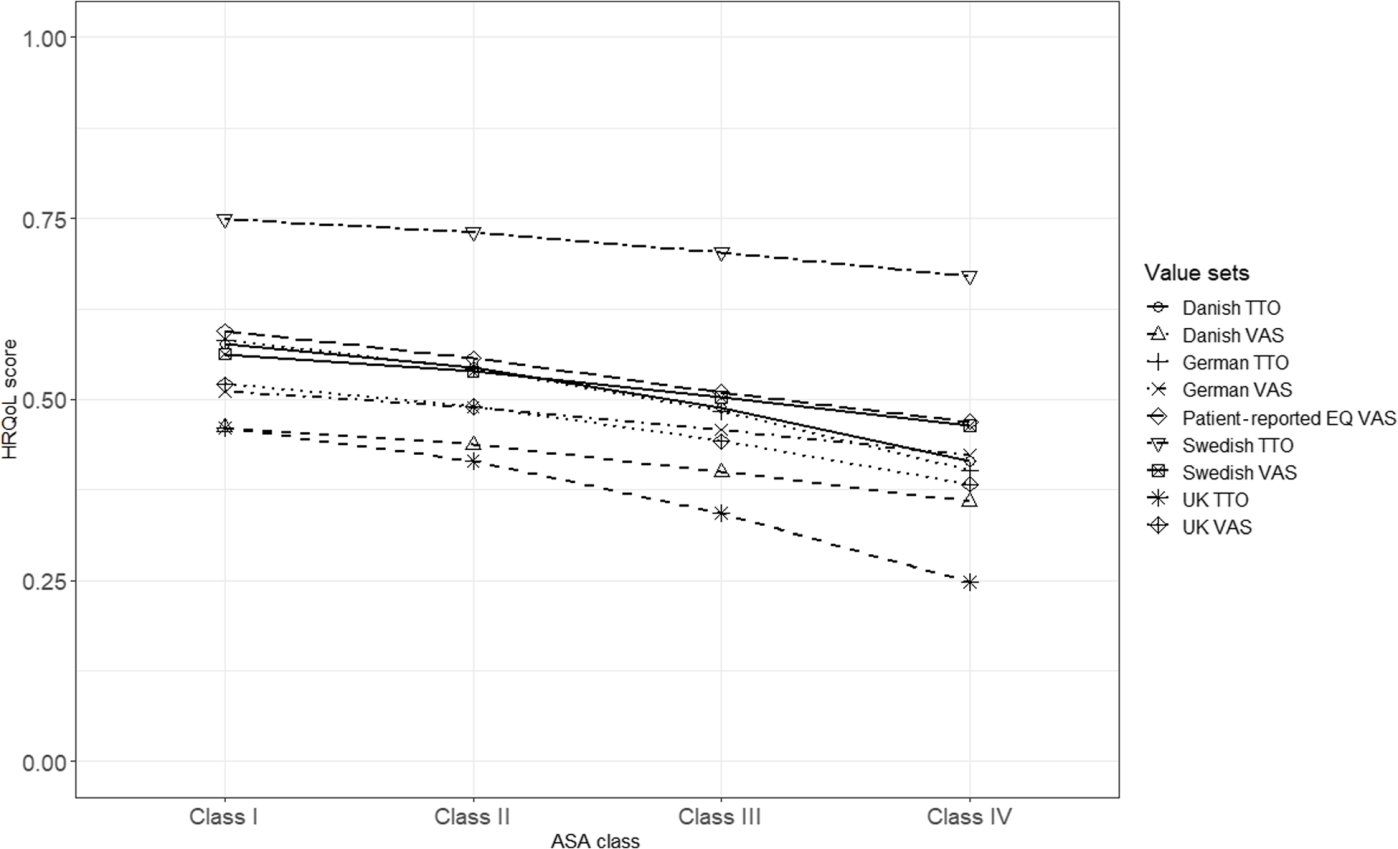
Mean EQ-5D indices and patient-reported EQ VAS score by ASA class, preoperative (*n* = 69,290)

**Fig. 2:**
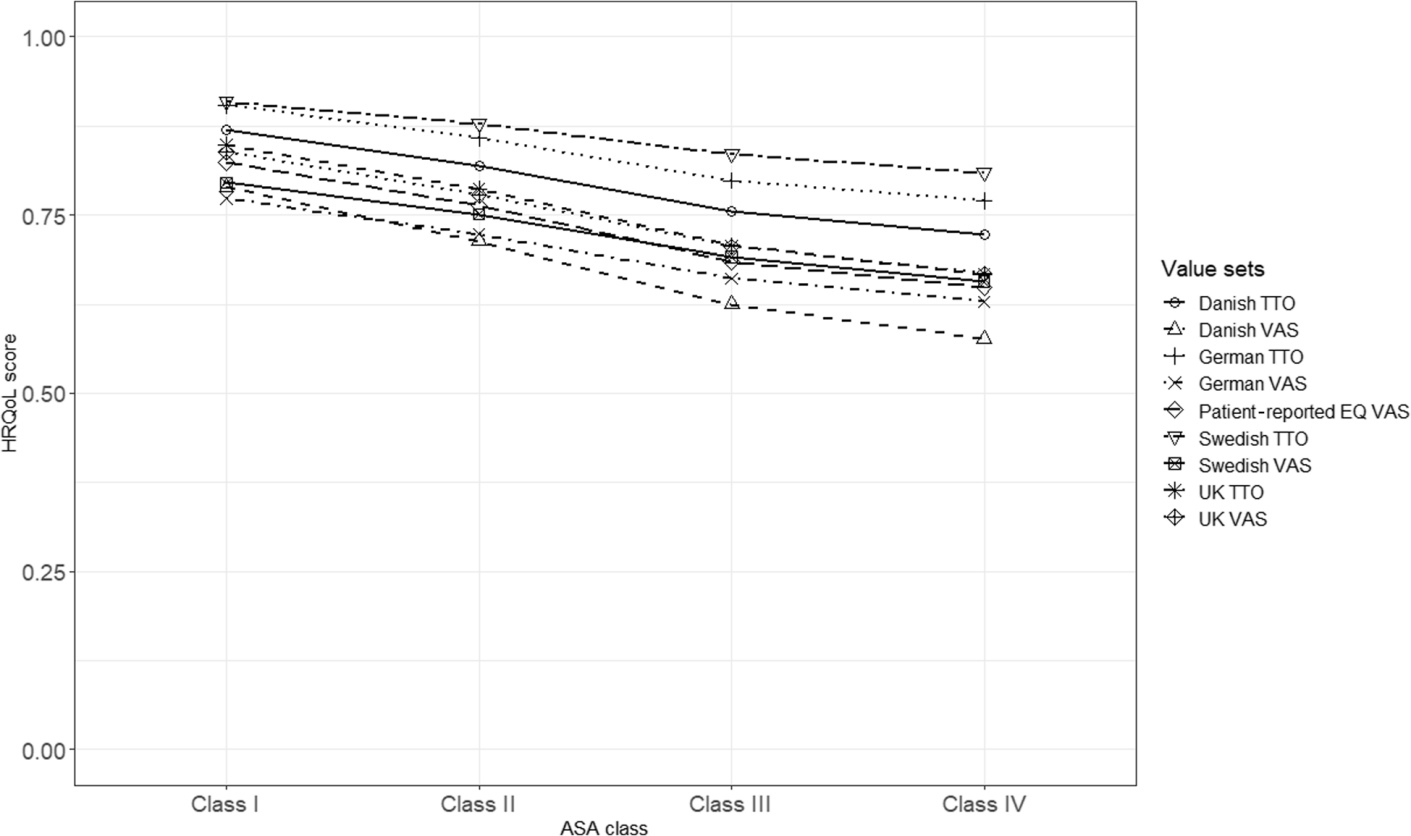
Mean patient-reported EQ VAS score and EQ-5D indices by ASA class 1 year postoperatively (*n* = 69,290)

Mean EQ-5D indices based on all the value sets differed among ASA classes with lower indices for more severe ASA levels, pre- and 1 year postoperatively. The Swedish TTO had the highest mean index in all the four ASA classes compared to the other value sets preoperatively and 1 year postoperatively. Among the VAS value sets, the Swedish and UK value sets showed the highest mean indices pre- and 1 year postoperatively, respectively. In the 1 year postoperative follow-up, the VAS value sets and the hypothetical TTO value sets showed values closer to the Swedish TTO as compared to preoperative mean EQ-5D indices (Figs. 1, 2).

Mean differences in EQ-5D indices among ASA classes were statistically significant based on one-way ANOVA. Variations in EQ-5D indices of 1–2% preoperatively and 3–4.5% 1 year postoperatively were explained by ASA classes (Table 2). Post hoc analysis using Bonferroni adjusted p-values showed that all of the pairwise comparisons between ASA classes had statistically significant differences in EQ-5D indices preoperatively. One year postoperatively, the only comparisons containing no statistically significant differences involved mean indices in ASA class III and ASA IV. These were the hypothetical TTO value sets (Additional file 2, Tables S3-S4).

**Table 2:** One-way ANOVA test of abilities of value sets to differentiate among ASA classes pre- and 1 year postoperatively (*n* = 69,290)

Follow-up		Value sets							
		Swedish VAS	German VAS	Danish^**a**^ VAS	UK^**a**^ VAS	Swedish TTO	German^**a**^ TTO	Danish^**a**^ TTO	UK^**a**^ TTO
**Preoperative**									
Mean	Overall	0.54	0.49	0.44	0.49	0.73	0.54	0.54	0.42
	Class I	0.56	0.51	0.46	0.52	0.75	0.58	0.58	0.46
	Class II	0.54	0.49	0.44	0.49	0.73	0.54	0.54	0.42
	Class III	0.50	0.46	0.40	0.44	0.70	0.48	0.49	0.34
	Class IV	0.46	0.42	0.36	0.38	0.67	0.40	0.41	0.25
Effect size [Eta squared (η^2^)]		0.015	0.014	0.019	0.015	0.016	0.012	0.015	0.014
*P*-value		< 0.001	< 0.001	< 0.001	< 0.001	< 0.001	< 0.001	< 0.001	< 0.001
**1-year postoperative**									
Mean	Overall	0.75	0.73	0.72	0.78	0.88	0.86	0.82	0.79
	Class I	0.80	0.77	0.79	0.84	0.91	0.90	0.87	0.85
	Class II	0.75	0.72	0.71	0.78	0.88	0.86	0.82	0.79
	Class III	0.69	0.66	0.62	0.70	0.84	0.80	0.76	0.71
	Class IV	0.66	0.63	0.58	0.67	0.81	0.77	0.72	0.67
Effect size [Eta squared (η^2^)]		0.043	0.042	0.044	0.040	0.043	0.032	0.037	0.035
*P*-value		< 0.001	< 0.001	< 0.001	< 0.001	< 0.001	< 0.001	< 0.001	< 0.001

^a^ Hypothetical perspective

### Predictive effects of ASA class on HRQoL score

OLS models showed that ASA class predicted HRQoL when using patient-reported EQ VAS score and EQ-5D indices both pre- and 1 year postoperatively, regardless of value sets used. In all the models, the coefficients of ASA class II, III and IV showed decrements in HRQoL scores that were consistent in magnitude and direction (sign). Preoperatively, the UK TTO followed by the German and Danish TTO value sets showed the highest decrements in each ASA class among the models of all the value sets. One year postoperatively, the Danish VAS value set followed by the UK VAS and TTO value sets showed the highest decrements in the EQ-5D index across all the ASA classes. In comparison, the Swedish TTO had the lowest decrements preoperatively followed by the German VAS and 1 year postoperatively followed by the German TTO value set (Tables 3, 4).

**Table 3:** OLS models on the predictive effects of ASA classes on HRQoL score preoperatively (*n* = 69,290)

**Model components**	**Model estimates**									
	**Patient-reported EQ VAS**		**Swedish VAS**		**German VAS**		**Danish VAS**^**b**^		**UK VAS**^**b**^	
	**β**	**RSE**^**a**^	**β**	**RSE**^**a**^	**β**	**RSE**^**a**^	**Β**	**RSE**^**a**^	**β**	**RSE**^**a**^
Intercept	0.594^*^	0.0017	0.562^*^	0.0011	0.510^*^	0.0010	0.461^*^	0.0010	0.520^*^	0.0014
ASA class II	−0.038^*^	0.0020	−0.024^*^	0.0014	− 0.022^*^	0.0012	−0.024^*^	0.0012	−0.030^*^	0.0017
ASA class III	−0.084^*^	0.0028	−0.060^*^	0.0019	−0.053^*^	0.0018	−0.061^*^	0.0017	−0.078^*^	0.0025
ASA class IV	−0.125^*^	0.0151	−0.099^*^	0.0100	−0.088^*^	0.0095	−0.102^*^	0.0104	− 0.138^*^	0.0135
R^2^		0.0138		0.0145		0.0136		0.0188		0.0154
MAE		0.184		0.129		0.117		0.108		0.179
Normalized MAE		0.184		0.180		0.165		0.092		0.167
RMSE		0.222		0.153		0.140		0.138		0.195
Normalized RMSE		0.222		0.214		0.197		0.108		0.182
Spearman Rank correlation		0.129		0.112		0.162		0.132		0.125
**Model components**	**Swedish TTO**		**German TTO**^**b**^		**Danish TTO**^**b**^		**UK TTO**^**b**^			
	**β**	**RSE**^**a**^	**β**	**RSE**^**a**^	**β**	**RSE**^**a**^	**Β**	**RSE**^**a**^		
Intercept	0.749^*^	0.0008	0.581^*^	0.0020	0.577^*^	0.0016	0.460^*^	0.0023		
ASA class II	−0.018^*^	0.0010	−0.038^*^	0.0025	−0.033^*^	0.0020	−0.045^*^	0.0027		
ASA class III	−0.046^*^	0.0014	−0.098^*^	0.0035	−0.089^*^	0.0030	−0.118^*^	0.0039		
ASA Class IV	−0.079^*^	0.0079	−0.179^*^	0.0190	−0.163^*^	0.0174	−0.213^*^	0.0215		
R^2^		0.0156		0.0119		0.0150		0.0138		
MAE		0.094		0.269		0.201		0.293		
Normalized MAE		0.149		0.223		0.124		0.184		
RMSE		0.114		0.278		0.227		0.311		
Normalized RMSE		0.181		0.231		0.140		0.195		
Spearman Rank correlation		0.114		0.125		0.141		0.116		

*MAE* mean absolute error, *RMSE* root mean square error, *RSE* robust standard error

^*^P < 0.001

^a^Heteroskedasticity-consistent standard error;

^b^Hypothetical perspective

**Table 4:** OLS models on the predictive effect of ASA class on HRQoL score 1 year postoperatively (*n* = 69,290)

**Model components**	**Model estimates**									
	**Patient-reported EQ VAS**		**Swedish VAS**		**German VAS**		**Danish VAS**^**b**^		**UK VAS**^**b**^	
	**β**	**RSE**^**a**^	**β**	**RSE**^**a**^	**β**	**RSE**^**a**^	**β**	**RSE**^**a**^	**β**	**RSE**^**a**^
Intercept	0.824^*^	0.0013	0.795^*^	0.0010	0.773^*^	0.0011	0.790^*^	0.0012	0.838^*^	0.0014
ASA class II	−0.062^*^	0.0016	−0.044^*^	0.0012	−0.050^*^	0.0014	−0.076^*^	0.0015	−0.060^*^	0.0017
ASA class III	−0.140^*^	0.0025	−0.104^*^	0.0020	−0.112^*^	0.0021	−0.165^*^	0.0024	−0.132^*^	0.0026
ASA Class IV	−0.175^*^	0.0148	−0.138^*^	0.0119	− 0.145^*^	0.0121	−0.213^*^	0.0142	−0.170^*^	0.0150
R^2^		0.0474		0.0430		0.0423		0.0444		0.0402
MAE		0.153		0.122		0.138		0.207		0.162
Normalized MAE		0.153		0.170		0.194		0.178		0.151
RMSE		0.194		0.151		0.165		0.237		0.200
Normalized RMSE		0.194		0.211		0.232		0.203		0.186
Spearman Rank correlation		0.288		0.275		0.205		0.236		0.177
**Model components**	**Swedish TTO**		**German TTO**^**b**^		**Danish TTO**^**b**^		**UK TTO**^**b**^			
	**β**	**RSE**^**a**^	**β**	**RSE**^**a**^	**β**	**RSE**^**a**^	**β**	**RSE**^**a**^		
Intercept	0.909^*^	0.0007	0.903^*^	0.0011	0.870^*^	0.0012	0.848^*^	0.0015		
ASA class II	−0.030^*^	0.0009	−0.046^*^	0.0014	−0.051^*^	0.0015	−0.062^*^	0.0019		
ASA class III	−0.073^*^	0.0014	−0.105^*^	0.0024	−0.115^*^	0.0024	−0.141^*^	0.0031		
ASA Class IV	− 0.100^*^	0.0085	− 0.133^*^	0.0152	− 0.147^*^	0.0142	−0.181^*^	0.0186		
R^2^		0.0431		0.0318		0.0367		0.0348		
MAE		0.084		0.121		0.134		0.164		
Normalized MAE		0.133		0.101		0.083		0.111		
RMSE		0.106		0.179		0.182		0.229		
Normalized RMSE		0.169		0.148		0.112		0.247		
Spearman Rank correlation		0.164		0.124		0.156		0.187		

*MAE* mean absolute error; *RMSE* root mean square error, *RSE* robust standard error

^*^P < 0.001

^a^Heteroskedasticity-consistent standard error;

^b^Hypothetical perspective

In the adjusted models, which controlled for demographic, clinical and PRO variables, ASA class still predicted HRQoL, both pre- and 1 year postoperatively. In all the models, decrements in HRQoL were consistent with the ASA class (the higher the ASA class the larger the decrement). However, the magnitudes of decrement in all the models were lower than in the unadjusted models for each value set in both pre- and postoperative follow-ups (Additional file 2, Table S5; Table 5).

**Table 5:** Adjusted OLS models on the predictive effects of ASA class on HRQoL score based on the value sets 1 year postoperatively (*n* = 69,290)

Model components	Value sets/EQ VAS								
	Patient-reported EQ VAS	Swedish VAS	German VAS	Danish VAS^**h**^	UK VAS^**h**^	Swedish TTO	German TTO^**h**^	Danish TTO^**h**^	UK TTO^**h**^
Intercept	0.860^***^	0.834^***^	0.834^***^	0.880^***^	0.903^***^	0.932^***^	0.952^***^	0.917^***^	0.907^***^
ASA class ^a^									
Class II	−0.028^***^	−0.015^***^	− 0.015^***^	− 0.024^***^	− 0.020^***^	− 0.010^***^	− 0.015^***^	− 0.018^***^	− 0.022^***^
Class III	− 0.066^***^	− 0.040^***^	− 0.037^***^	−0.054^***^	− 0.047^***^	−0.029^***^	− 0.038^***^	−0.043^***^	− 0.054^***^
Class IV	−0.092^***^	− 0.066^***^	−0.060^***^	− 0.087^***^	−0.074^***^	− 0.051^***^	−0.060^***^	− 0.068^***^	−0.084^***^
Age group ^b^									
50–59	0.011^***^	0.010^***^	0.006^**^	0.012^**^	0.009^**^	0.007^***^	0.004	0.010^***^	0.010^**^
60–69	0.029^***^	0.021^***^	0.015^***^	0.019^***^	0.019^***^	0.016^***^	0.013^***^	0.022^***^	0.026^***^
70–79	0.021^***^	0.020^***^	0.013^***^	0.013^***^	0.017^***^	0.016^***^	0.013^***^	0.022^***^	0.026^***^
80–98	−0.006	0.002	−0.004	−0.020^***^	− 0.005	0.002	− 0.001	0.005	0.006^***^
Sex ^c^									
Female	−0.010^***^	−0.018^***^	− 0.020^***^	−0.030^***^	− 0.024^***^	−0.012^***^	− 0.017^***^	−0.022^***^	− 0.025^***^
BMI category ^d^									
Normal weight	0.025^***^	0.024^***^	0.021^***^	0.034^***^	0.029^***^	0.018^***^	0.022^***^	0.027^***^	0.032^***^
Overweight	0.026^***^	0.024^***^	0.020^***^	0.033^***^	0.028^***^	0.017^***^	0.020^**^	0.026^***^	0.032^***^
Obese class I	0.018^*^	0.017^**^	0.012^*^	0.021^**^	0.019^**^	0.012^**^	0.011	0.019^**^	0.022^**^
Obese class II	0.009	0.012^*^	0.008	0.014	0.013	0.008^*^	0.003	0.014^*^	0.015
Obese class III	−0.007	−0.005	−0.007	−0.003	−0.003	−0.005	−0.011	− 0.003	−0.005
Laterality ^e^									
Left	−0.001	−0.002	− 0.002	−0.002	− 0.001	−0.001	0.0001	−0.001	− 0.0006
Charnley class ^f^									
Class B	− 0.063^***^	−0.053^***^	− 0.073^***^	−0.126^***^	− 0.097^***^	−0.033^***^	− 0.062^***^	−0.075^***^	− 0.091^***^
Class C	−0.157^***^	− 0.146^***^	−0.173^***^	− 0.256^***^	−0.199^***^	− 0.098^***^	−0.149^***^	− 0.166^***^	−0.202^***^
Hip pain level ^g^									
Very mild	−0.085^***^	−0.073^***^	− 0.093^***^	−0.143^***^	− 0.106^***^	−0.048^***^	− 0.063^***^	−0.084^***^	− 0.100^***^
Mild	−0.167^***^	− 0.138^***^	−0.150^***^	− 0.194^***^	−0.156^***^	− 0.096^***^	−0.120^***^	− 0.138^***^	−0.171^***^
Moderate	−0.285^***^	−0.257^***^	− 0.259^***^	−0.278^***^	− 0.319^***^	−0.184^***^	− 0.366^***^	−0.342^***^	− 0.447^***^
Severe	−0.372^***^	− 0.331^***^	−0.317^***^	− 0.346^***^	−0.414^***^	− 0.254^***^	−0.498^***^	− 0.511^***^	−0.621^***^
Adjusted R^2^	0.360	0.460	0.503	0.486	0.455	0.441	0.384	0.431	0.415
MAE	0.121	0.083	0.092	0.143	0.117	0.058	0.088	0.100	0.123
Normalized MAE	0.121	0.116	0.129	0.123	0.109	0.092	0.073	0.062	0.077
RMSE	0.159	0.114	0.119	0.174	0.151	0.081	0.143	0.140	0.178
Normalized RMSE	0.159	0.159	0.167	0.149	0.140	0.129	0.118	0.086	0.112
Spearman’s Rank correlation	0.598	0.683	0.701	0.696	0.692	0.688	0.687	0.679	0.682

Reference group: ^a^ class I;^b^ < 50; ^c^Male; ^d^ underweight; ^e^ right; ^f^ class A; ^g^ none; ^h^hypothetical perspective; ^*^P-value< 0.05; ^**^P-value< 0.01;^***^P-value< 0.001

The smallest non-statistically significant *p*-value = 0.059; *MAE* mean absolute error, *RMSE* root mean square error

Generally, R^2^ increased in the models for the 1-year postoperative follow-up compared to the preoperative ones. EQ-5D index based on the Danish VAS value set had the largest R^2^ followed by the Swedish TTO, preoperatively. One year postoperatively, the patient-reported EQ VAS score and the Danish VAS value set showed the highest R^2^ (Tables 3, 4). In the adjusted models, the UK TTO had the highest adjusted R^2^ followed by the German TTO preoperatively and the German VAS followed by the Danish VAS 1 year postoperatively. Similar to the unadjusted model, adjusted R^2^ increased from preoperative to 1-year postoperative follow-up (Additional file 2, Table S5; Table 5).

In terms of the difference between observed and predicted EQ-5D indices, the Swedish TTO had the smallest MAEs as well as RMSE both pre- and 1 year postoperatively. However, the normalized versions of these error measures, which take the difference in the range of the EQ-5D index in the value sets into consideration, showed that preoperatively the Danish VAS has the lowest MAE and RMSE followed by the Danish and Swedish TTO value sets. One year postoperatively, the Danish TTO showed the lowest normalized MAE and RMSE followed by the German and UK TTO value sets (Tables 3, 4).

In the adjusted models as well, MAE and RMSE values of the Swedish TTO were the lowest both pre- and postoperatively. However, the Danish VAS and TTO value sets demonstrated the lowest normalized MAE and RMSE preoperatively followed by the UK value sets and the Swedish TTO. One year postoperatively, the Danish TTO followed by the German TTO value set showed the lowest normalized MAE and RMSE (Additional file 2, Table S5; Table 5).

As to the correlation between predicted and observed EQ-5D indices, the German VAS and the patient-reported EQ VAS had the highest values preoperatively and 1 year postoperatively, respectively. In the adjusted models, the German and UK TTO value sets had the highest correlation coefficients preoperatively while the German VAS had the highest correlation coefficient 1 year postoperatively (Additional file 2, Table S5; Table 5).

### Findings of the 6-year postoperative follow-up

In the 6-year postoperative follow-up, the reported problems in the EQ-5D-3L dimensions showed a similar pattern to that of the 1-year postoperative follow-up, however, the proportion of problems reported showed an increase (Additional file 2, Table S6). The mean EQ-5D indices among the value sets decreased in the 6-year postoperative follow-up (Additional file 2, Table S7). The mean indices in the different ASA classes showed a similar pattern as the preoperative and 1-year postoperative follow-up (Additional file 2, Table S8). In the OLS models, patient-reported EQ VAS score, consistent levels of decrements from ASA II to ASA IV similar to the preoperative and 1-year postoperative follow-ups were shown (Additional file 2, Table S9). However, in the adjusted models, all value sets exhibited lower decrements in HRQoL score in ASA class IV than in class III (Additional file 2, Table S10). All the findings related to the 6-year postoperative follow-up can be found in Additional file 2 (Tables S6-S12) and Additional file 1 (Figures S8-S13).

## Discussion

In the present study, we assessed the abilities of EQ-5D-3L value sets from Sweden, Germany, Denmark, and the UK to differentiate across ASA classes and in showing whether ASA classes predict HRQoL. The findings showed that all the value sets differentiated across ASA classes in terms of HRQoL. Moreover, we found that ASA class predict HRQoL score in both unadjusted and adjusted OLS models, regardless of the value set.

Lower EQ-5D indices were observed in the more severe ASA classes across value sets. Specifically, the Swedish TTO showed higher EQ-5D indices in each group compared to the other value sets in the preoperative data. This difference became much lower compared to the hypothetical TTO value sets and slightly lower compared to the VAS value sets in the 1-year postoperative follow-up. This may be related to differences between the experience-based Swedish TTO and the hypothetical TTO value sets (German, Danish, and the UK) in valuing severe health states. The latter value sets have much lower indices in severe health states compared to the Swedish TTO. Meanwhile, healthier states were valued closer to the high values of the Swedish TTO value set.

Patient-reported EQ VAS scores and all the EQ-5D value sets demonstrated the predictive ability of ASA classes on HRQoL score in a consistent manner. A study assessing the relationship between ASA class and functional recovery after surgery for hip fracture showed better walking ability, mental health status and general health for ASA class I/II compared to ASA class III. The findings are, generally, similar to the present study in that ASA class was shown to be related to functional outcomes and HRQoL [25]. The findings in the present study were also similar to that of another which assessed ASA class as a predictor of HRQoL, among other factors, measured using different instruments including the EQ-5D after surgical intervention for hip fracture. Prediction of HRQoL by ASA class and larger decrements in higher classes were reported, similar to the present study [26]. The present study provided information on how HRQoL results may vary depending on the value sets chosen to be used to calculate the EQ-5D index.

Among the models, decrements were the highest for the hypothetical TTO value sets preoperatively and for the Danish VAS and both value sets from the UK 1 year postoperatively. This could be explained by the wider EQ-5D index intervals of the five hypothetical value sets in the present study compared to the other value sets. Their EQ-5D indices for full health are set at 1 and they also contain negative indices in the most severe health states, which likely contributed to higher decrements shown in different ASA classes.

All the models demonstrated larger R^2^ values postoperatively compared to preoperative models, indicating predictive ability in explaining the variation in HRQoL score by ASA class. In the postoperative follow-up, the patient-reported EQ VAS score and the VAS value sets showed a larger R^2^. The Swedish TTO showed closer R^2^ value to these value sets than the other TTO value sets. The larger R^2^ values among the VAS value sets may partly be attributed to their development through the VAS method.

As to prediction error, the Swedish TTO had the smallest MAE and RMSE across pre- and 1-year postoperative follow-ups in both the unadjusted and adjusted models. Normalized versions of these measures indicated that the Danish VAS followed by the Danish and Swedish TTO among the preoperative models and the Danish TTO among the 1-year postoperative models showed the smallest prediction errors. Among the possible reasons could be the lower valuations of severe health states in these value sets which could contribute to more differentiated values across ASA classes.

In interpreting the findings from the present study, a possible limitation that needs to be taken into consideration relates to the difference in the timing of the valuation studies which produced the value sets compared in the present study. The time difference which ranged as wide as between 1997 and 2014 could introduce variation may not be explained by methodological, perspective or country differences [15, 16, 31–34]. Another possible limitation of the present study involves variation in the models employed in the development of the value sets compared which may influence the differences among the value sets beside the valuations respondents provided to the health states they valued. The weights to health states resulting from different models and the number of parameters resulting from the models could have influenced the EQ-5D indices. In addition, the comparisons made in this study should be understood in the context that anchoring for dead at zero was not set for the VAS value sets from Sweden and Germany.

In relation to investigating the predictive ability of ASA class on HRQoL score, the present study provided information on the characteristics of the different value sets in terms of explained variation, the magnitude of decrements and level of prediction error among others in the context of patients in SHAR. This will be useful information to add to current knowledge on the relationship of ASA classes with HRQoL score. One of the ways the findings of the present study can be used could involve applying the information that ASA classes, among other factors, are predictors of HRQoL among patients. This information can be used to determine patients in higher ASA classes and assess ways of providing care which can help improve their HRQoL. The study also indicated that the prediction on HRQoL by ASA class was fairly similar across the different value sets employed to calculate HRQoL.

## Conclusion

In the present study, differences in HRQoL score across ASA classes was demonstrated by all the value sets employed. In line with this, ASA classes were shown to predict HRQoL score with all value sets. Hence, the study revealed that the ASA classification of the physical status of patients preoperatively predicts their HRQoL score across pre- and postoperative follow-ups. The information on the ASA class of patients can be useful in tailoring the care provided to patients with the aim of eventual improvement in their HRQoL after THR. In addition, the study found that levels of the predictive performance of ASA class on HRQoL were only slightly varying by type of EQ-5D-3L value set used, making the relationship in general fairly robust against changes among the value sets investigated.

## Supplementary information

**Additional file 1: Figure S1.** Sampling procedure. **Figure S2.** Patient-reported EQ VAS score by ASA class pre- and 1 year postoperatively (*n* = 69,290). **Figure S3.** Patient-reported EQ VAS score pre- and 1 year postoperatively (*n* = 69,290). **Figure S4.** Patient-reported EQ VAS score by Charnley category pre- and 1 year postoperatively (*n* = 69,290). **Figure S5.** Patient-reported EQ VAS score by hip pain, pre- and 1 year postoperatively (*n* = 69,290). **Figure S6.** Distributions of EQ-5D indices based on the value sets preoperatively (*n* = 69,290). **Figure S7.** Distributions of EQ-5D indices based on the value sets 1 year postoperatively (*n* = 69,290). **Figure S8.** Patient-reported EQ VAS score 6 years postoperatively (*n* = 21,305). **Figure S9.** Patient-reported EQ VAS score by ASA class 6 years postoperatively (*n* = 21,305). **Figure S10.** Patient-reported EQ VAS score by hip pain level 6 years postoperatively (*n* = 21,305). **Figure S11.** Patient-reported EQ VAS score by Charnley class, 6 years postoperatively (*n* = 21,305). **Figure S12.** Distributions of EQ-5D indices based on the value sets 6 years postoperatively (*n* = 21,305). **Figure S13.** Mean EQ-5D indices and patient-reported EQ VAS score by ASA class, 6 years postoperatively (*n* = 21,305)

**Additional file 2: Table S1.** Prevalence of reported problems in the EQ-5D dimensions by ASA class pre- and 1 year postoperatively (*n* = 69,290). **Table S2.** Mean and median EQ-5D indices based on the different value sets among THR patients by ASA class pre- and 1 year postoperatively (*n* = 69,290). **Table S3.** Post hoc Bonferroni adjusted p-values of pairwise tests of the one-way ANOVA test for abilities of value sets to differentiate among ASA classes pre- and 1 year postoperatively (*n* = 69,290). **Table S4.** Kruskal Wallis rank sum Test to assess abilities of value sets to differentiate HRQoL by ASA class (*n* = 69,290). **Table S5.** Adjusted OLS models on the predictive effects of ASA class on HRQoL score based on the value sets preoperatively (*n* = 69,290). **Table S6.** Prevalence of reported problems in the EQ-5D dimensions by ASA class, 6 years postoperatively (*n* = 21,305). **Table S7.** Mean EQ-5D indices based on the different value sets 6 years postoperatively (*n* = 21,305). **Table S8.** One-way ANOVA test of abilities of value sets to differentiate among ASA classes 6 years postoperatively (*n* = 21,305). **Table S9.** OLS models of predictive effect of ASA class on HRQoL score by value set 6 years postoperatively (*n* = 21,305). **Table S10.** Adjusted OLS models ASA class on HRQoL score by value sets 6 years postoperatively (*n* = 21,305). **Table S11** Post hoc Bonferroni adjusted *p*-values of pairwise tests of the one-way ANOVA test for abilities of value sets to differentiate among ASA classes 6 years postoperatively (*n* = 21,305). **Table S12.** Kruskal Wallis rank sum Test to assess abilities of value sets to differentiate HRQoL by ASA class, 6 years postoperatively (*n* = 21,305)

## Data Availability

The dataset analysed during this study is not publicly available as the study was approved on the ground of ensuring the confidentiality of data of patients included in the study. However, the data are available to authorised personnel from the Swedish Hip Arthroplasty Register subject to approval by the register and an ethics review committee.

## Abbreviations

ANOVA: Analysis of variance
ASA: American Society of Anaesthesiologists
BMI: Body mass index
EQ VAS: EuroQol visual analogue scale
HRQoL: Health-related quality of life
MAE: Mean absolute error
PROs: Patient-reported outcome
RMSE: Root mean square error
SHAR: Swedish Hip Arthroplasty Register
THR: Total hip replacement
TTO: Time trade-off
VAS: Visual analogue scale
