# Supplementary material for: Predictive ability of the American Society of Anaesthesiologists physical status classification system on health-related quality of life of patients after total hip replacement: comparisons across eight EQ-5D-3L value sets
Source: BMC Musculoskelet Disord. 2020 Jul 6;21:441. doi: 10.1186/s12891-020-03399-8 (PMC7339530; doi:10.1186/s12891-020-03399-8)
Supplement: Supplementary file 1 — Additional file 1: Figure S1. Sampling procedure. Figure S2. Patient-reported EQ VAS score by ASA class pre- and 1 year postoperatively (n = 69,290). Figure S3. Patient-reported EQ VAS score pre- and 1 year postoperatively (n = 69,290). Figure S4. Patient-reported EQ VAS score by Charnley category pre- and 1 year postoperatively (n = 69,290). Figure S5. Patient-reported EQ VAS score by hip pain, pre- and 1 year postoperatively (n = 69,290). Figure S6. Distributions of EQ-5D indices based on the value sets preoperatively (n = 69,290). Figure S7. Distributions of EQ-5D indices based on the value sets 1 year postoperatively (n = 69,290). Figure S8. Patient-reported EQ VAS score 6 years postoperatively (n = 21,305). Figure S9. Patient-reported EQ VAS score by ASA class 6 years postoperatively (n = 21,305). Figure S10. Patient-reported EQ VAS score by hip pain level 6 years postoperatively (n = 21,305). Figure S11. Patient-reported EQ VAS score by Charnley class, 6 years postoperatively (n = 21,305). Figure S12. Distributions of EQ-5D indices based on the value sets 6 years postoperatively (n = 21,305). Figure S13. Mean EQ-5D indices and patient-reported EQ VAS score by ASA class, 6 years postoperatively (n = 21,305) [file 12891_2020_3399_MOESM1_ESM.pdf]

Additional file 1

Title: Predictive ability of the American Society of Anaesthesiologists physical status classification system on health-related quality of life of patients after total hip replacement: Comparisons across eight EQ-5D-3L value sets

Journal: BMC Musculoskeletal Disorders

Authors: Fitsum Sebsibe Teni, Kristina Burström, Jenny Berg, Reiner Leidl and Ola Rolfson

Corresponding author
Fitsum Sebsibe Teni
Health Outcomes and Economic Evaluation Research Group, Stockholm Centre for Healthcare Ethics, Department of Learning Informatics, Management and Ethics (LIME), Karolinska Institutet, Stockholm, Sweden
Email: fitsum.teni@ki.se

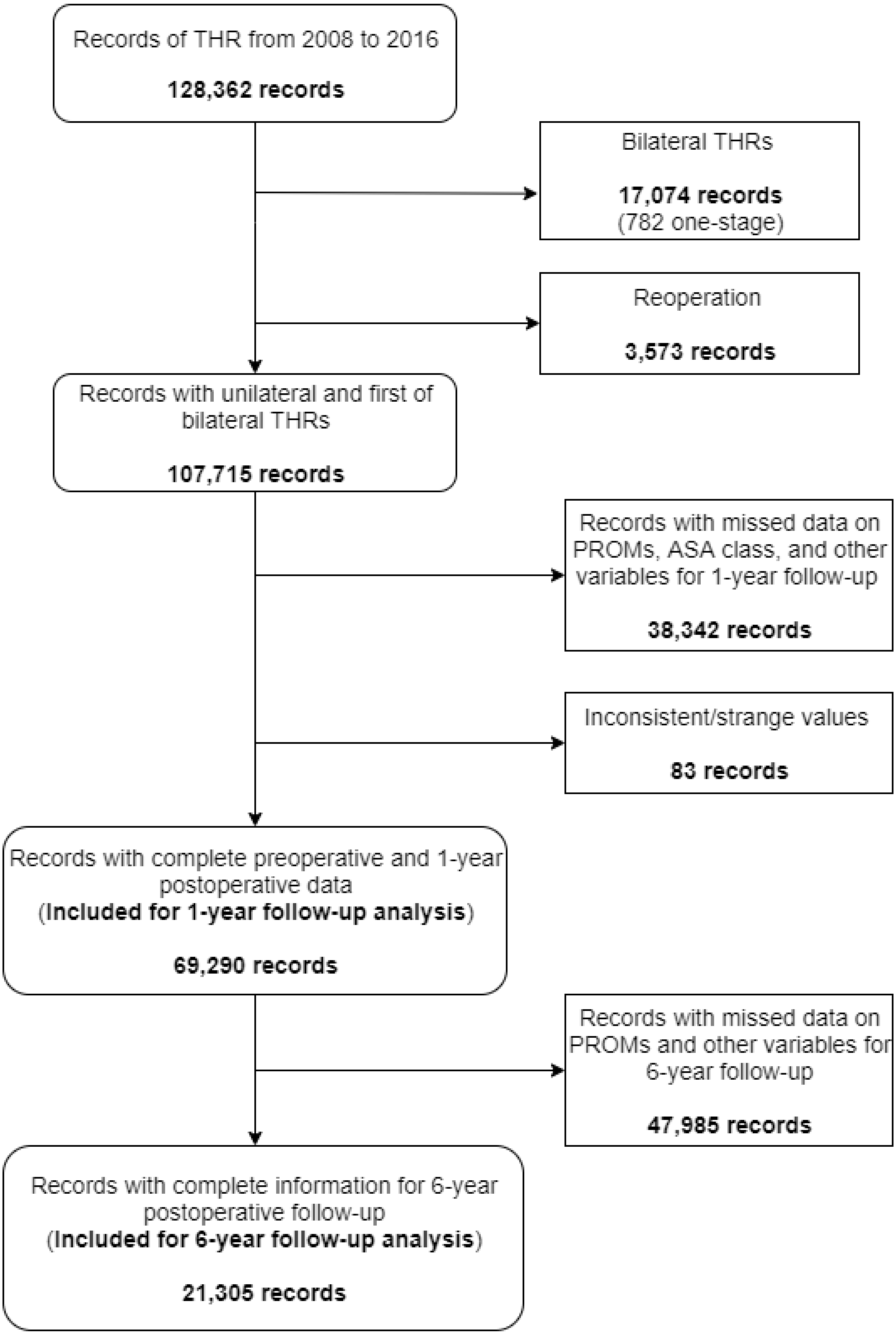

Figure S1: Sampling procedure

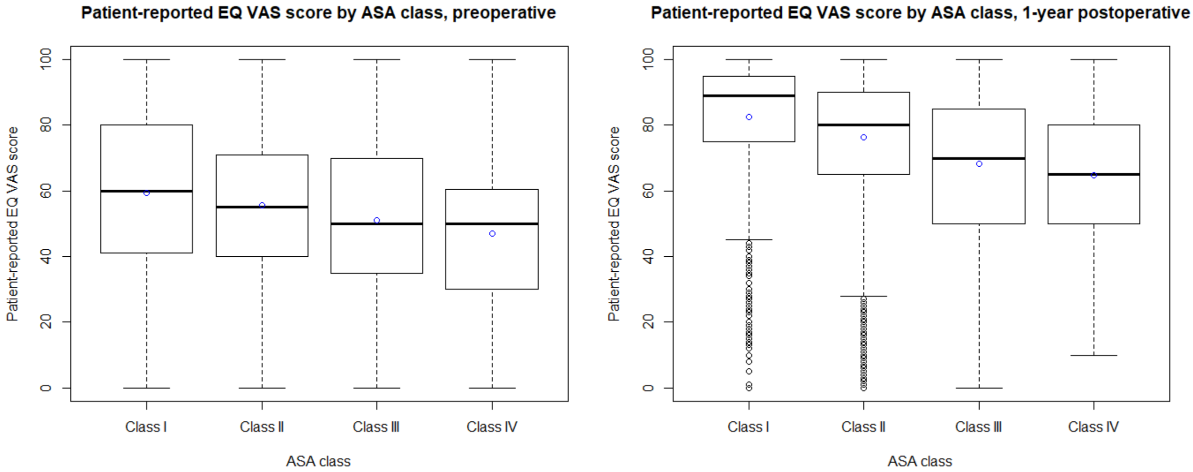

Figure S2: Patient-reported EQ VAS score by ASA class pre- and 1 year postoperatively (n=69,290)

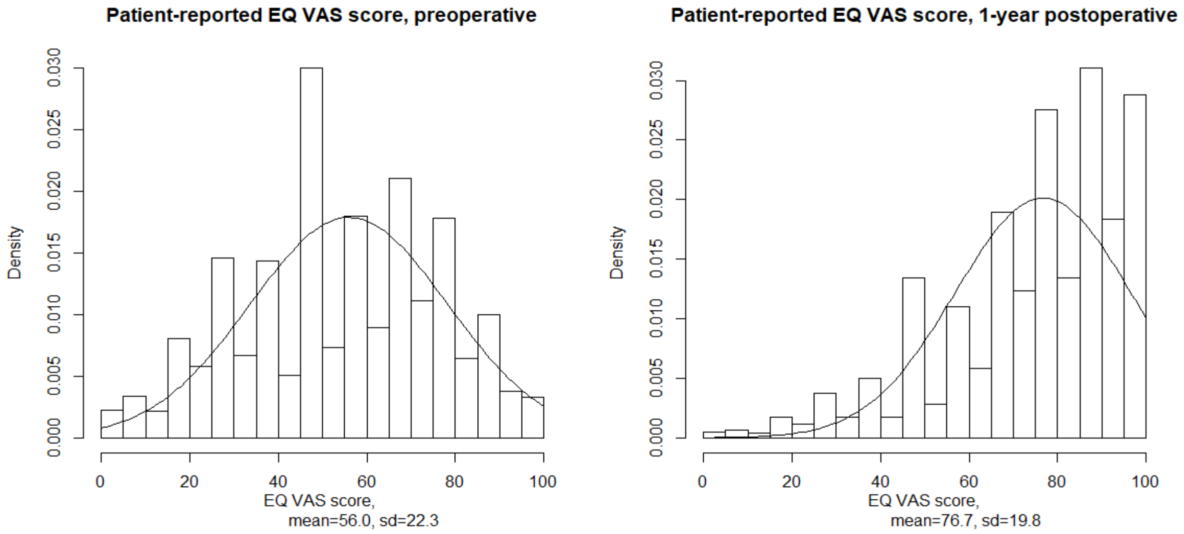

Figure S3: Patient-reported EQ VAS score pre- and 1 year postoperatively (n= 69,290)

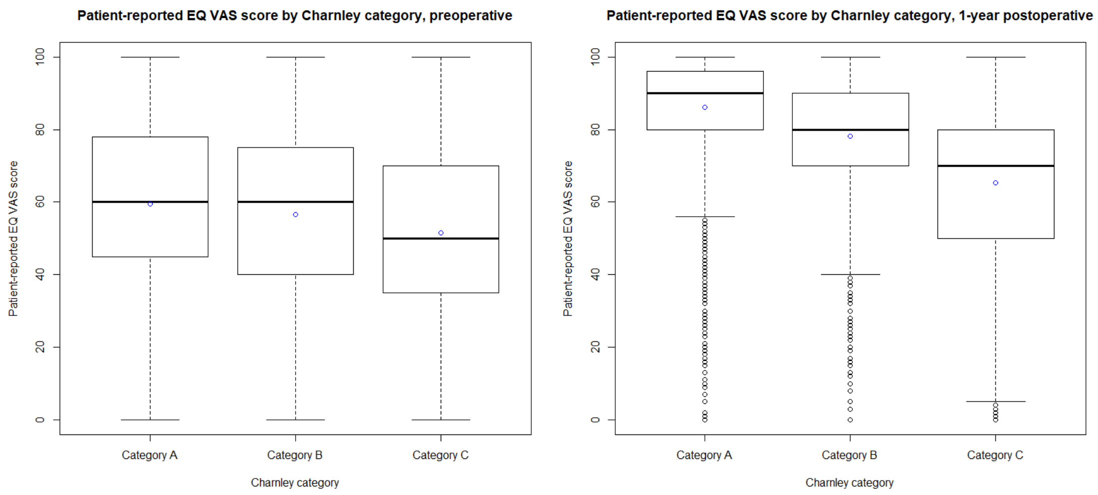

Figure S4: Patient-reported EQ VAS score by Charnley category pre- and 1 year postoperatively (n= 69,290)

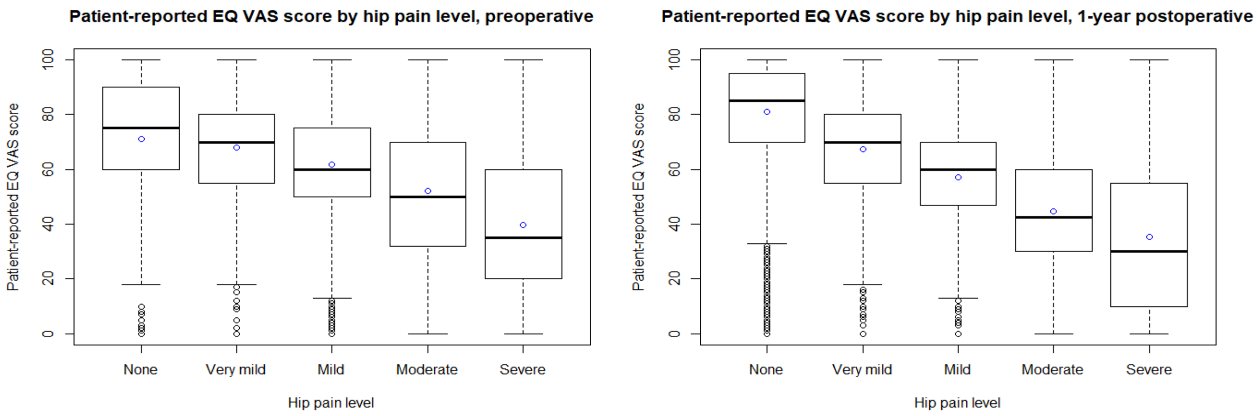

Figure S5: Patient-reported EQ VAS score by hip pain, pre- and 1 year postoperatively (n=69,290)

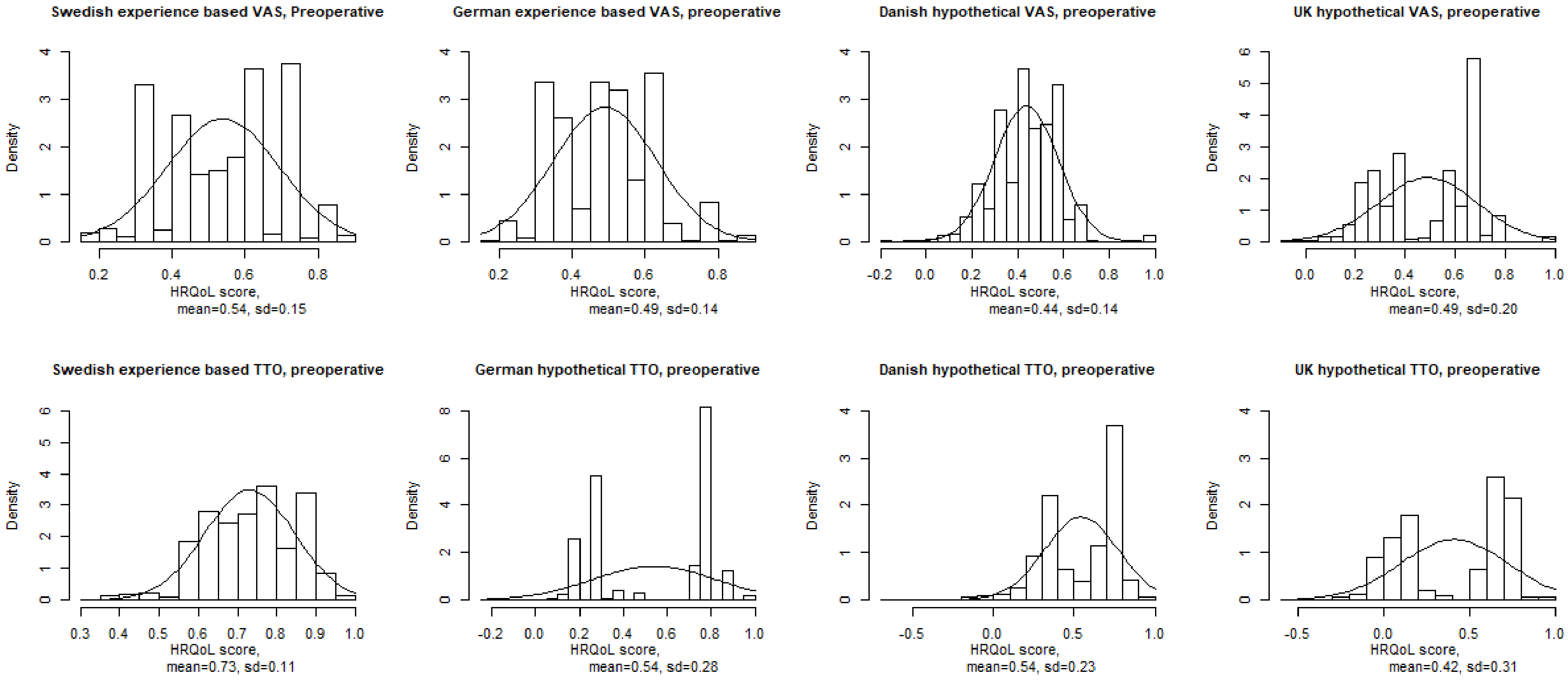

Figure S6: Distributions of EQ-5D indices based on the value sets preoperatively (n=69,290)

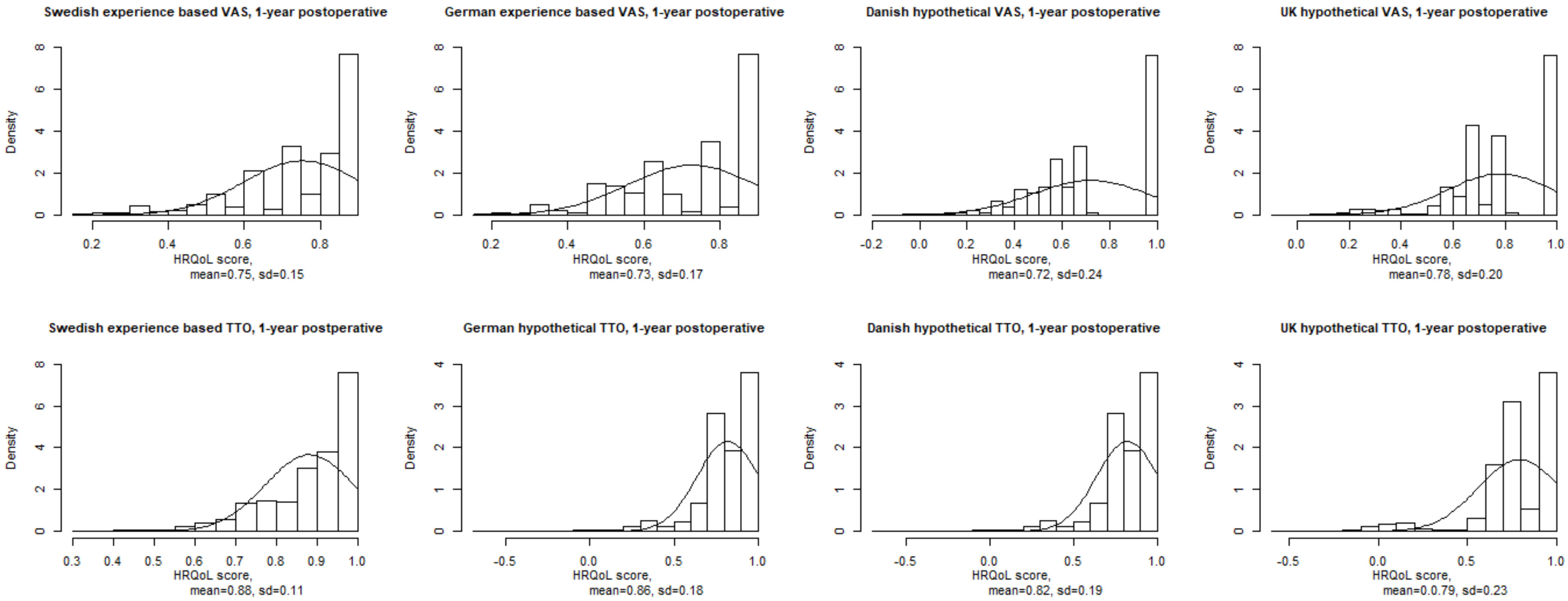

Figure S7: Distributions of EQ-5D indices based on the value sets 1 year postoperatively (n=69,290)

Findings of the 6-year postoperative follow-up

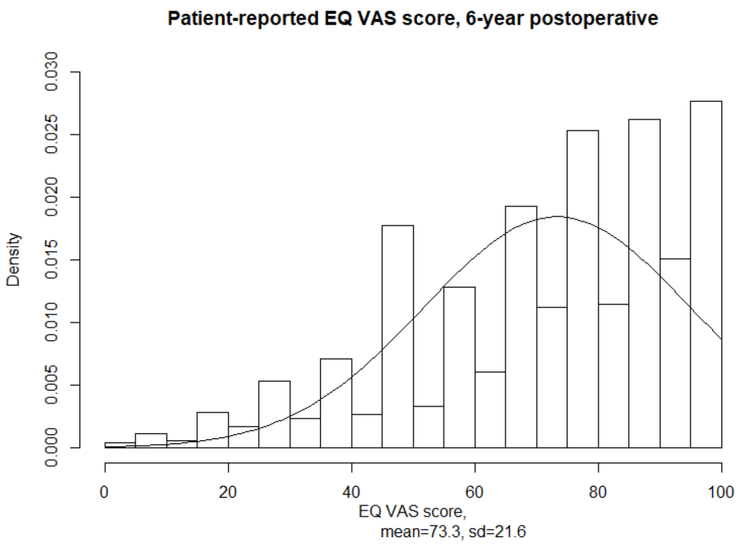

Figure S8: Patient-reported EQ VAS score 6 years postoperatively (n=21,305)

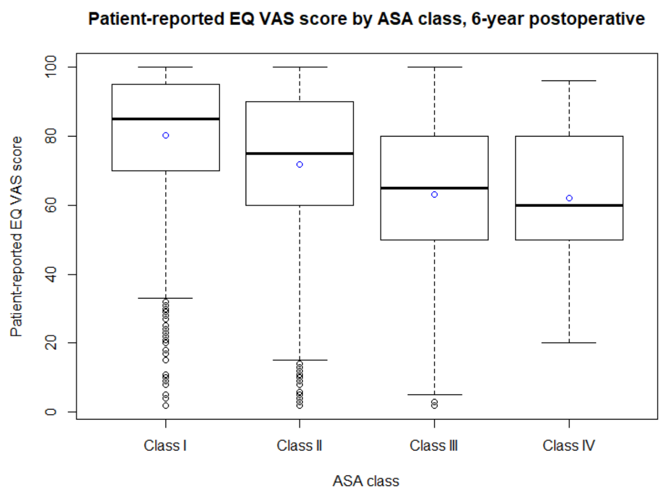

Figure S9: Patient-reported EQ VAS score by ASA class 6 years postoperatively (n=21,305)

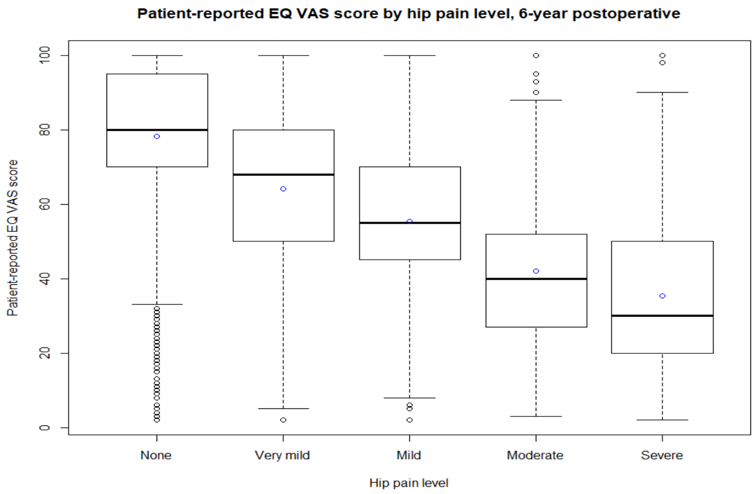

Figure S10: Patient-reported EQ VAS score by hip pain level 6 years postoperatively (n=21,305)

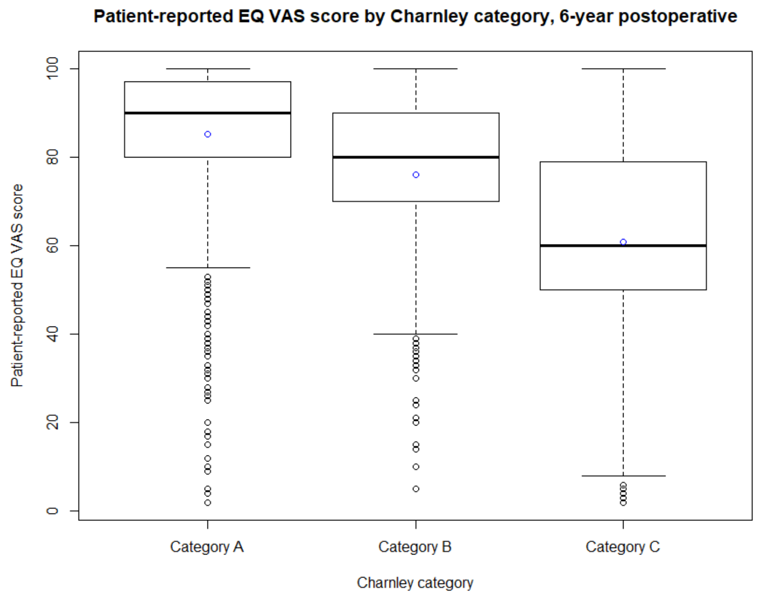

Figure S11: Patient-reported EQ VAS score by Charnley class, 6 years postoperatively (n=21,305)

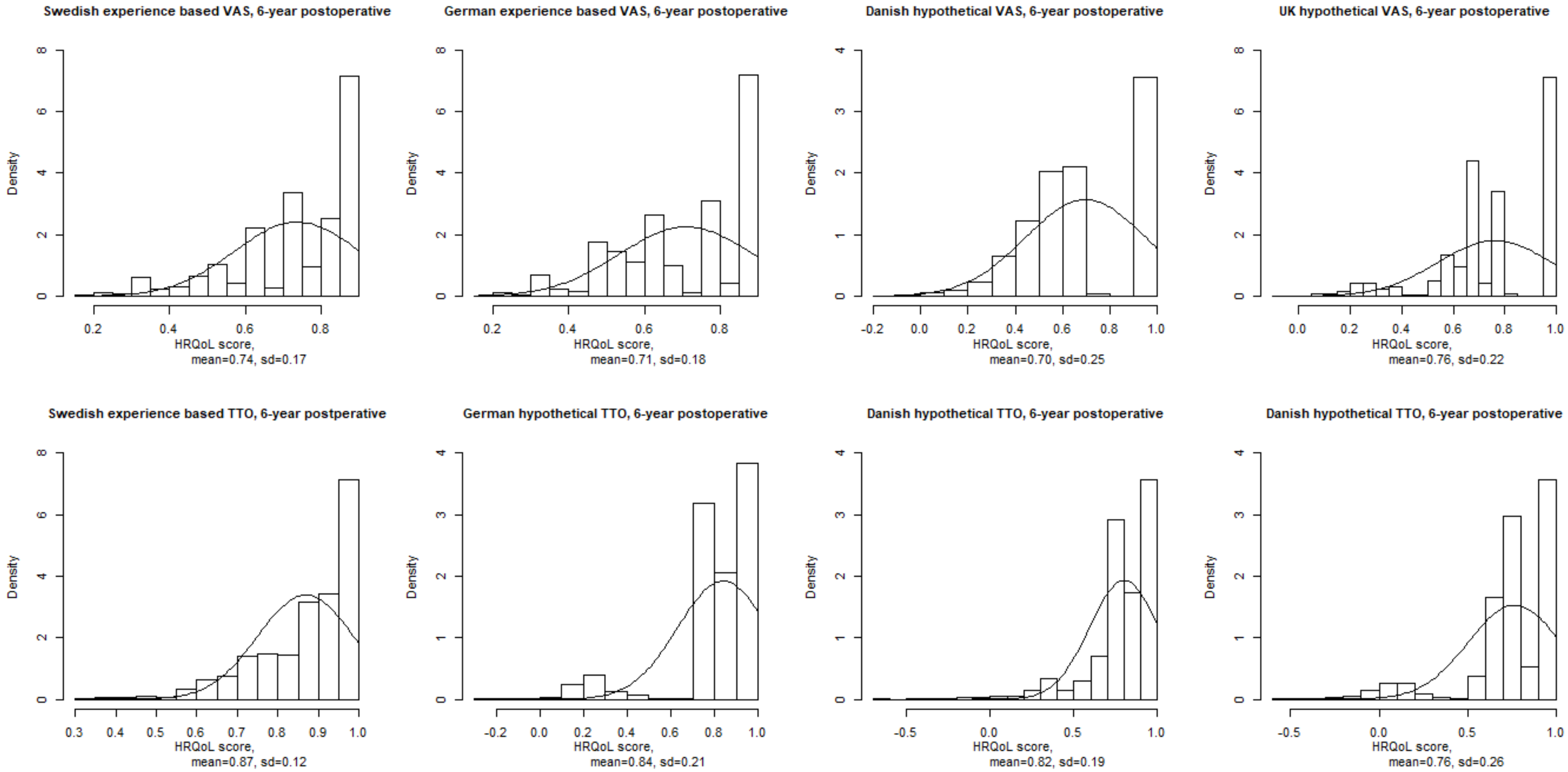

Figure S12: Distributions of EQ-5D indices based on the value sets 6 years postoperatively (n=21,305)

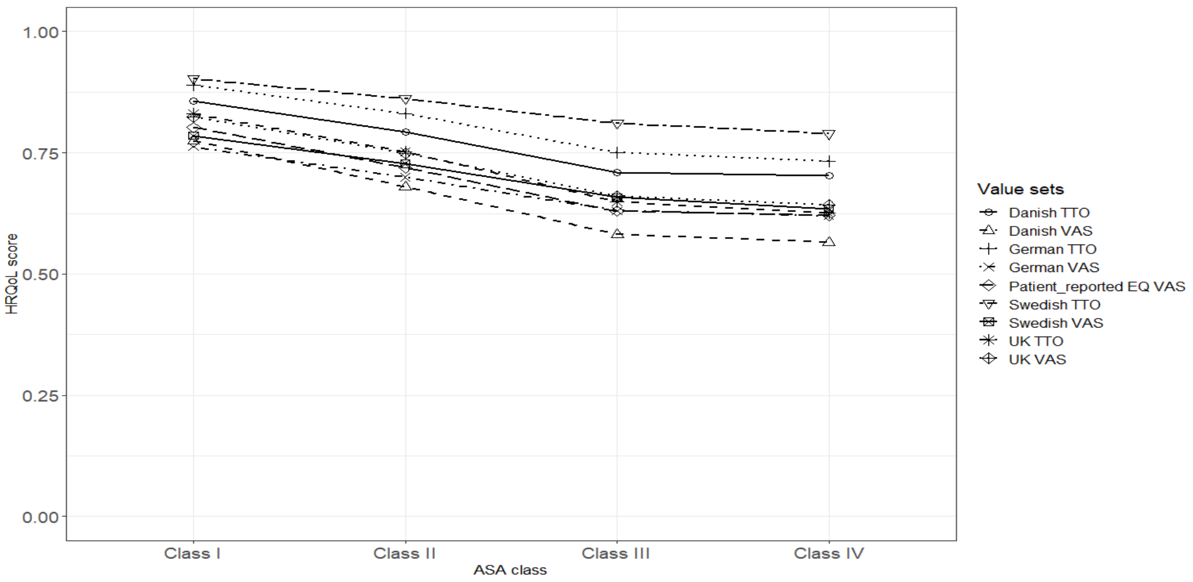

Figure S13: Mean EQ-5D indices and patient-reported EQ VAS score by ASA class, 6 years postoperatively (n= 21,305)
