# Supplementary material for: Predictive ability of the American Society of Anaesthesiologists physical status classification system on health-related quality of life of patients after total hip replacement: comparisons across eight EQ-5D-3L value sets
Source: BMC Musculoskelet Disord. 2020 Jul 6;21:441. doi: 10.1186/s12891-020-03399-8 (PMC7339530; doi:10.1186/s12891-020-03399-8)
Supplement: Supplementary file 2 — Additional file 2: Table S1. Prevalence of reported problems in the EQ-5D dimensions by ASA class pre- and 1 year postoperatively (n = 69,290). Table S2. Mean and median EQ-5D indices based on the different value sets among THR patients by ASA class pre- and 1 year postoperatively (n = 69,290). Table S3. Post hoc Bonferroni adjusted p-values of pairwise tests of the one-way ANOVA test for abilities of value sets to differentiate among ASA classes pre- and 1 year postoperatively (n = 69,290). Table S4. Kruskal Wallis rank sum Test to assess abilities of value sets to differentiate HRQoL by ASA class (n = 69,290). Table S5. Adjusted OLS models on the predictive effects of ASA class on HRQoL score based on the value sets preoperatively (n = 69,290). Table S6. Prevalence of reported problems in the EQ-5D dimensions by ASA class, 6 years postoperatively (n = 21,305). Table S7. Mean EQ-5D indices based on the different value sets 6 years postoperatively (n = 21,305). Table S8. One-way ANOVA test of abilities of value sets to differentiate among ASA classes 6 years postoperatively (n = 21,305). Table S9. OLS models of predictive effect of ASA class on HRQoL score by value set 6 years postoperatively (n = 21,305). Table S10. Adjusted OLS models ASA class on HRQoL score by value sets 6 years postoperatively (n = 21,305). Table S11 Post hoc Bonferroni adjusted p-values of pairwise tests of the one-way ANOVA test for abilities of value sets to differentiate among ASA classes 6 years postoperatively (n = 21,305). Table S12. Kruskal Wallis rank sum Test to assess abilities of value sets to differentiate HRQoL by ASA class, 6 years postoperatively (n = 21,305) [file 12891_2020_3399_MOESM2_ESM.pdf]

Additional file 2

Title: Predictive ability of the American Society of Anaesthesiologists physical status classification system on health-related quality of life of patients after total hip replacement: Comparisons across eight EQ-5D-3L value sets

Journal: BMC Musculoskeletal Disorders

Authors: Fitsum Sebsibe Teni, Kristina Burström, Jenny Berg, Reiner Leidl and Ola Rolfson

Corresponding author
Fitsum Sebsibe Teni
Health Outcomes and Economic Evaluation Research Group, Stockholm Centre for Healthcare Ethics, Department of Learning Informatics, Management and Ethics (LIME), Karolinska Institutet, Stockholm, Sweden
Email: fitsum.teni@ki.se

Table S1: Prevalence of reported problems in the EQ-5D dimensions by ASA class pre- and 1 year postoperatively (n=69,290)

	ASA class							
	Class I		Class II		Class III		Class IV	
**EQ-5D dimension**	**%**	**n**	**%**	**n**	**%**	**n**	**%**	**n**
**Preoperative**								
Mobility								
No problem	10.5	1,868	7.3	3011	4.7	462	4.6	10
Some problems	89.4	15,926	92.5	38370	94.5	9223	92.7	203
Confined to bed	0.2	28	0.3	111	0.7	72	2.7	6
Self-care								
No problem	82.3	14,667	77.3	32089	68.2	6655	57.5	126
Some problems	17.2	3,059	21.9	9069	30.0	2929	37.9	83
Unable to	0.5	96	0.8	334	1.8	173	4.6	10
Usual activities								
No problem	43.0	7,664	39.1	16243	31.4	3065	21.0	46
Some problems	49.0	8,725	51.1	21208	54.1	5282	58.4	128
Unable to	8.0	1,433	9.7	4041	14.5	1410	20.5	45
Pain/discomfort								
No problem	1.5	276	1.5	635	1.3	124	1.4	3
Moderate problems	62.1	11,069	56.7	23509	48.4	4722	37.0	81
Extreme problems	36.3	6,477	41.8	17348	50.3	4911	61.6	135
Anxiety/depression								
No problem	63.5	11,314	57.9	24007	52.0	5074	49.8	109
Moderate problems	33.9	6,042	38.8	16100	43.1	4208	43.4	95
Extreme problems	2.6	466	3.3	1385	4.9	475	6.8	15
**1-year postoperative**								
Mobility								
No problem	75.3	13,426	59.6	24,722	41.5	4,048	34.7	76
Some problems	24.6	4,389	40.3	16,725	58.2	5,683	64.4	141
Confined to bed	0.0	7	0.1	45	0.3	26	0.9	2
Self-care								
No problem	96.1	17,131	93.0	38,588	85.9	8,382	79.0	173
Some problems	3.7	659	6.5	2,696	12.9	1,255	18.3	40
Unable to	0.2	32	0.5	208	1.2	120	2.7	6
Usual activities								
No problem	85.8	15,292	78.0	32,384	64.1	6,258	49.3	108
Some problems	13.3	2,376	20.2	8,401	31.9	3,108	45.2	99
Unable to	0.9	154	1.7	707	4.0	391	5.5	12
Pain/discomfort								
No problem	54.7	9,744	43.2	17,912	33.4	3,256	29.7	65
Moderate problems	42.9	7,640	52.2	21,661	58.9	5,745	62.6	137
Extreme problems	2.5	438	4.6	1,919	7.7	756	7.8	17
Anxiety/depression								
No problem	85.1	15,174	78.3	32,485	69.1	6,744	65.3	143
Moderate problems	13.9	2,485	20.4	8,445	28.5	2,778	32.4	71
Extreme problems	0.9	163	1.4	562	2.4	235	2.3	5

Table S2: Mean and median EQ-5D indices based on the different value sets among THR patients by ASA class pre- and 1 year postoperatively (n=69,290)

ASA class	Swedish VAS	German VAS	Danish VAS	UK VAS	Swedish TTO	German TTO	Danish TTO	UK TTO
**Preoperative**								
Class I								
Mean	0.56	0.51	0.46	0.52	0.75	0.58	0.58	0.46
SD	0.15	0.14	0.14	0.19	0.11	0.27	0.22	0.30
Median	0.60	0.54	0.48	0.60	0.77	0.79	0.66	0.62
Class II								
Mean	0.54	0.49	0.44	0.49	0.73	0.54	0.54	0.42
SD	0.15	0.14	0.14	0.20	0.11	0.28	0.23	0.31
Median	0.57	0.49	0.43	0.57	0.74	0.70	0.66	0.59
Class III								
Mean	0.50	0.46	0.40	0.44	0.70	0.48	0.49	0.34
SD	0.15	0.14	0.14	0.20	0.12	0.28	0.24	0.32
Median	0.50	0.46	0.42	0.39	0.71	0.29	0.44	0.20
Class IV								
Mean	0.46	0.42	0.36	0.38	0.67	0.40	0.41	0.25
SD	0.15	0.13	0.16	0.20	0.12	0.28	0.26	0.32
Median	0.45	0.37	0.36	0.36	0.67	0.26	0.39	0.16
Overall								
Mean	0.54	0.49	0.44	0.49	0.73	0.54	0.54	0.42
SD	0.15	0.14	0.14	0.20	0.12	0.28	0.23	0.31
Median	0.57	0.49	0.43	0.57	0.74	0.70	0.66	0.59
**1-year postoperative**								
Class I								
Mean	0.80	0.77	0.79	0.84	0.91	0.90	0.87	0.85
SD	0.13	0.15	0.23	0.18	0.09	0.15	0.16	0.20
Median	0.82	0.81	0.71	0.81	0.94	1.00	0.84	0.88
Class II								
Mean	0.75	0.72	0.71	0.78	0.88	0.86	0.82	0.79
SD	0.15	0.17	0.24	0.20	0.11	0.18	0.18	0.23
Median	0.82	0.76	0.70	0.76	0.94	0.89	0.82	0.80
Class III								
Mean	0.69	0.66	0.62	0.70	0.84	0.80	0.76	0.71
SD	0.17	0.18	0.24	0.22	0.12	0.22	0.21	0.27
Median	0.72	0.65	0.57	0.69	0.87	0.79	0.77	0.73
Class IV								
Mean	0.66	0.63	0.58	0.67	0.81	0.77	0.73	0.67
SD	0.18	0.18	0.24	0.22	0.13	0.22	0.21	0.28
Median	0.62	0.59	0.51	0.66	0.81	0.79	0.72	0.69
Overall								
Mean	0.75	0.73	0.72	0.78	0.88	0.86	0.82	0.79
SD	0.16	0.17	0.24	0.20	0.11	0.18	0.18	0.23
Median	0.82	0.76	0.70	0.76	0.94	0.89	0.82	0.80

Table S3: Post hoc Bonferroni adjusted p-values of pairwise tests of the one-way ANOVA test for abilities of value sets to differentiate among ASA classes pre- and 1 year postoperatively (n=69,290)

Follow-up	P-value							
	Swedish VAS	German VAS	Danish VAS[a]	UK VAS[a]	Swedish TTO	German TTO[a]	Danish TTO[a]	UK TTO[a]
**Preoperative**								
Class I, Class II	<0.001	<0.001	<0.001	<0.001	<0.001	<0.001	<0.001	<0.001
Class I, Class III	<0.001	<0.001	<0.001	<0.001	<0.001	<0.001	<0.001	<0.001
Class I, Class IV	<0.001	<0.001	<0.001	<0.001	<0.001	<0.001	<0.001	<0.001
Class II, Class III	<0.001	<0.001	<0.001	<0.001	<0.001	<0.001	<0.001	<0.001
Class II, Class IV	<0.001	<0.001	<0.001	<0.001	<0.001	<0.001	<0.001	<0.001
Class III, Class IV	0.001	0.002	<0.001	<0.001	<0.001	<0.001	<0.001	<0.001
**1-year postoperative**								
Class I, Class II	<0.001	<0.001	<0.001	<0.001	<0.001	<0.001	<0.001	<0.001
Class I, Class III	<0.001	<0.001	<0.001	<0.001	<0.001	<0.001	<0.001	<0.001
Class I, Class IV	<0.001	<0.001	<0.001	<0.001	<0.001	<0.001	<0.001	<0.001
Class II, Class III	<0.001	<0.001	<0.001	<0.001	<0.001	<0.001	<0.001	<0.001
Class II, Class IV	<0.001	<0.001	<0.001	<0.001	<0.001	<0.001	<0.001	<0.001
Class III, Class IV	0.006	0.022	0.018	0.034	0.002	0.130	0.065	0.065

[a]Hypothetical perspective

Table S4: Kruskal Wallis rank sum Test to assess abilities of value sets to differentiate HRQoL by ASA class (n=69,290)

		Kruskal Wallis X^2							
**Follow-up**	**ASA class**	**Swedish VAS**	**German VAS**	**Danish [a] VAS**	**UK VAS**	**Swedish TTO**	**German [a] TTO**	**Danish [a] TTO**	**UK TTO**
Preoperative	Class I<br>Class II<br>Class III<br>Class IV	1014.1*	941.4*	1230.7*	1136.1*	1035.2*	1014.3*	1063.8*	1095.1*
1-year postoperative	Class I<br>Class II<br>Class III<br>Class IV	3055.8*	2925.0*	3253.1*	3006.2*	3162.2*	2790.6*	2926.6*	2833.9*

[a]hypothetical perspective
*P-value<0.001

Table S5: Adjusted OLS models on the predictive effects of ASA class on HRQoL score based on the value sets preoperatively (n=69,290)

Model components	Value sets/EQ VAS								
	Patient-reported EQ VAS	Swedish VAS	German VAS	Danish VAS[h]	UK VAS[h]	Swedish TTO	German TTO[h]	Danish TTO[h]	UK TTO[h]
Intercept	0.735***	0.661***	0.621***	0.549***	0.656***	0.816***	0.778***	0.720***	0.670***
ASA class [a]									
Class II	-0.030***	-0.018***	-0.015***	-0.017***	-0.018***	-0.014***	-0.019***	-0.020***	-0.026***
Class III	-0.062***	-0.038***	-0.032***	-0.040***	-0.043***	-0.030***	-0.045***	-0.049***	-0.061***
Class IV	-0.077***	-0.052***	-0.043***	-0.058***	-0.066***	-0.043***	-0.076***	-0.079***	-0.098***
Age group [b]									
50-59	0.009*	0.009***	0.007**	0.001***	0.008*	0.007***	0.005	0.010*	0.011*
60-69	0.029***	0.033***	0.026***	0.028***	0.030***	0.025***	0.027***	0.036***	0.043***
70-79	0.042***	0.042***	0.033***	0.033***	0.038***	0.032***	0.034***	0.045***	0.055***
80-98	0.033***	0.030***	0.024***	0.018***	0.020***	0.022***	0.014**	0.028***	0.030***
Sex [c]									
Female	-0.037*	-0.018***	-0.017***	-0.009***	-0.018***	-0.010***	-0.025***	0.024***	-0.032***
BMI category [d]									
Normal weight	0.009	0.018**	0.013*	0.019**	0.017*	0.013**	0.012	0.019*	0.022
Overweight	0.009	0.016**	0.011*	0.017***	0.014	0.011*	0.007	0.017*	0.018
Obese class I	-0.004	0.008	0.004	0.008	0.002	0.005	-0.008	0.005	0.0005
Obese class II	-0.004	0.00004	-0.003	-0.002	-0.013	-0.002	-0.030**	-0.010	-0.023
Obese class III	-0.009	-0.010	-0.011	-0.016*	-0.031**	-0.012*	-0.049***	-0.034**	-0.050***
Laterality [e]									
Left	-0.0006	-0.0006	-0.0006	-0.0005	-0.0004	-0.0005	-0.003	-0.0004	-0.0005
Charnley class [f]									
Class B	-0.020***	-0.015***	-0.014***	-0.016***	-0.020***	-0.012***	-0.025***	-0.022***	-0.030***
Class C	-0.056***	-0.038***	-0.034***	-0.039***	-0.042***	-0.028***	-0.046***	-0.046***	-0.059***
Hip pain level [g]									
Very mild	-0.032***	-0.008	-0.017***	-0.024***	-0.006	-0.004	-0.008	0.003	0.006
Mild	-0.087***	-0.055***	-0.061***	-0.063***	-0.051***	-0.038***	-0.046***	-0.044***	-0.059***
Moderate	-0.173***	-0.172***	-0.174***	-0.145***	-0.229***	-0.121***	-0.322***	-0.251***	-0.359***
Severe	-0.281***	-0.252***	-0.243***	-0.221***	-0.340***	-0.185***	-0.471***	-0.403***	-0.542***
Adjusted R^2	0.138	0.282	0.300	0.219	0.338	0.268	0.357	0.350	0.360
MAE	0.171	0.105	0.095	0.094	0.126	0.079	0.180	0.140	0.199
Normalized MAE	0.171	0.146	0.134	0.080	0.118	0.125	0.149	0.086	0.125
RMSE	0.207	0.131	0.118	0.123	0.160	0.098	0.225	0.184	0.250
Normalized RMSE	0.207	0.182	0.166	0.105	0.149	0.156	0.186	0.113	0.157
Spearman's Rank correlation	0.353	0.542	0.550	0.477	0.563	0.508	0.568	0.573	0.573

Reference group: [a] Class I; [b] < 50; [c] Male; [d] Underweight; [e] Right; [f] Class A; [g] None; [h] hypothetical perspective; *P-value<0.05; **P-value<0.01; ***P-value<0.001

MAE: mean absolute error

RMSE; root mean square error

Findings of the 6-year postoperative follow-up

Table S6: Prevalence of reported problems in the EQ-5D dimensions by ASA class, 6 years postoperatively (n=21,305)

EQ-5D dimension	ASA class							
	Class I		Class II		Class III		Class IV	
	%	n	%	n	%	n	%	n
Mobility								
No problem	71.8	4,507	53.1	6,709	35.2	832	37.0	10
Some problems	28.0	1,759	46.5	5,877	63.2	1,495	63.0	17
Confined to bed	0.2	10	0.4	50	1.6	39	0.0	0
Self-care								
No problem	95.5	5,992	90.2	11,398	80.8	1,911	63.0	17
Some problems	4.0	251	8.6	1,081	15.8	374	29.6	8
Unable to	0.5	33	1.2	157	3.4	81	7.4	2
Usual activities								
No problem	85.5	5,365	73.5	9,291	57.3	1,355	44.4	12
Some problems	13.2	830	23.1	2,924	35.8	848	51.9	14
Unable to	1.3	81	3.3	421	6.9	163	3.7	1
Pain/discomfort								
No problem	52.7	3,306	39.8	5,024	29.9	707	33.3	9
Moderate problems	43.5	2,733	53.9	6,817	58.5	1,384	59.3	16
Extreme problems	3.8	237	6.3	795	11.6	275	7.4	2
Anxiety/depression								
No problem	83.0	5,208	73.6	9,301	63.4	1,499	59.3	16
Moderate problems	15.9	998	24.8	3,138	33.5	793	33.3	9
Extreme problems	1.1	70	1.6	197	3.1	74	7.4	2

Table S7: Mean EQ-5D indices based on the different value sets 6 years postoperatively (n=21,305)

ASA class	Value set							
	**Swedish VAS**	**German VAS**	**Danish VAS**	**UK VAS**	**Swedish TTO**	**German TTO**	**Danish TTO**	**UK TTO**
Class I								
Mean	0.78	0.76	0.77	0.82	0.90	0.89	0.86	0.83
SD	0.14	0.16	0.24	0.20	0.10	0.17	0.18	0.22
Median	0.82	0.79	0.70	0.77	0.94	1.00	0.82	0.85
Class II								
Mean	0.73	0.70	0.68	0.75	0.86	0.83	0.79	0.75
SD	0.17	0.18	0.25	0.22	0.12	0.21	0.20	0.26
Median	0.72	0.73	0.63	0.74	0.88	0.89	0.78	0.79
Class III								
Mean	0.66	0.63	0.58	0.66	0.81	0.75	0.71	0.65
SD	0.18	0.19	0.26	0.24	0.14	0.26	0.25	0.31
Median	0.70	0.65	0.57	0.69	0.83	0.79	0.76	0.72
Class IV								
Mean	0.64	0.62	0.56	0.64	0.79	0.73	0.70	0.62
SD	0.21	0.21	0.29	0.27	0.15	0.27	0.24	0.32
Median	0.60	0.54	0.45	0.62	0.77	0.79	0.70	0.68
Overall								
Mean	0.74	0.71	0.70	0.76	0.87	0.84	0.80	0.76
SD	0.17	0.18	0.25	0.22	0.12	0.21	0.21	0.26
Median	0.79	0.76	0.68	0.76	0.91	0.89	0.82	0.80

Table S8: One-way ANOVA test of abilities of value sets to differentiate among ASA classes 6 years postoperatively (n=21,305)

Follow-up		Value sets							
		Swedish VAS	German VAS	Danish[a] VAS	UK[a] VAS	Swedish TTO	German[a] TTO	Danish[a] TTO	UK[a] TTO
**6-year postoperative**									
	Class I	0.78	0.76	0.77	0.82	0.90	0.89	0.86	0.83
Mean	Class II	0.73	0.70	0.68	0.75	0.86	0.83	0.79	0.75
	Class III	0.66	0.63	0.58	0.66	0.81	0.75	0.71	0.65
	Class IV	0.64	0.62	0.56	0.64	0.79	0.73	0.70	0.62
Effect size [Eta squared (η^2)]		0.053*	0.051*	0.053*	0.049*	0.053*	0.039*	0.044*	0.043*

[a] Hypothetical perspective
*P-value < 0.001

Table S9: OLS models of predictive effect of ASA class on HRQoL score by value set 6 years postoperatively (n=21,305)

**Model components**	**Model estimates**									
	**Patient-reported EQ VAS**		**Swedish VAS**		**German VAS**		**Danish VAS[b]**		**UK VAS[b]**	
	**β**	**RSE[a]**	**β**	**RSE[a]**	**β**	**RSE[a]**	**β**	**RSE[a]**	**β**	**RSE[a]**
Intercept	0.802*	0.0024	0.785*	0.0018	0.762*	0.0020	0.774*	0.0030	0.824	0.0025
ASA class II	-0.083*	0.0031	-0.058*	0.0023	-0.063*	0.0025	-0.094*	0.0037	-0.075	0.0032
ASA class III	-0.172*	0.0053	-0.127*	0.0042	-0.132*	0.0043	-0.193*	0.0061	-0.163	0.0056
ASA class IV	-0.181*	0.0412	-0.151*	0.0396	-0.143*	0.0393	-0.209*	0.0556	-0.181	0.0509
R^2		0.0578		0.0527		0.0508		0.0532		0.0486
MAE		0.170		0.130		0.144		0.212		0.171
Normalized MAE		0.174		0.181		0.204		0.182		0.159
RMSE		0.210		0.161		0.172		0.247		0.216
Normalized RMSE		0.214		0.225		0.243		0.212		0.201
Spearman Rank correlation		0.160		0.227		0.222		0.192		0.264

**Model components**	**Swedish TTO**		**German TTO[b]**		**Danish TTO[b]**		**UK TTO[b]**	
	**β**	**RSE[a]**	**β**	**RSE[a]**	**β**	**RSE[a]**	**β**	**RSE[a]**
Intercept	0.902*	0.0012	0.889*	0.0022	0.856*	0.0022	0.831	0.0028
ASA class II	-0.040*	0.0016	-0.058*	0.0028	-0.064*	0.0029	-0.079	0.0036
ASA class III	-0.091*	0.0030	-0.139*	0.0057	-0.147*	0.0056	-0.182	0.0070
ASA Class IV	-0.113*	0.0289	-0.157*	0.0508	-0.153*	0.0445	-0.205	0.0614
R^2		0.0532		0.0386		0.0442		0.0425
MAE		0.091		0.207		0.147		0.182
Normalized MAE		0.144		0.172		0.090		0.114
RMSE		0.115		0.241		0.202		0.256
Normalized RMSE		0.182		0.200		0.124		0.160
Spearman Rank correlation		0.232		0.214		0.169		0.118

[a]Heteroskedasticity-consistent standard error;
[b]Hypothetical perspective
MAE: mean absolute error;
RSE: robust standard error;
RMSE: root mean square error;
*P<0.001

Table S10: Adjusted OLS models ASA class on HRQoL score by value sets 6 years postoperatively (n=21,305)

Model components	Value sets/EQ VAS								
	Patient-reported EQ VAS	Swedish VAS	German VAS	Danish[h] VAS	UK[h] VAS	Swedish TTO	German[h] TTO	Danish[h] TTO	UK[h] TTO
Intercept	0.861***	0.847***	0.839***	0.880***	0.914***	0.942***	0.967***	0.929***	0.924***
ASA class [a]									
Class II	-0.034***	-0.016***	-0.015***	-0.024***	-0.020***	-0.011***	-0.013***	-0.017***	-0.020***
Class III	-0.074***	-0.043***	-0.038***	-0.057***	-0.052***	-0.033***	-0.047***	-0.052***	-0.064***
Class IV	-0.050	-0.039	-0.016	-0.029	-0.034	-0.036*	-0.038	-0.027	-0.048
Age group [b]									
50-59	0.019**	0.010*	0.007	0.013*	0.011*	0.007*	0.007	0.012*	0.013
60-69	0.027***	0.016***	0.012**	0.018**	0.018***	0.012***	0.013*	0.020***	0.023***
70-79	-0.002	0.002	0.0002	-0.005	0.003	0.002	0.003	0.007	0.009
80-98	-0.044***	-0.041***	-0.037***	-0.071***	-0.054***	-0.032***	-0.046***	-0.042***	-0.054***
Sex [c]									
Female	-0.012***	-0.017***	-0.019***	-0.026***	-0.022***	-0.011***	-0.016***	-0.021***	-0.025***
BMI category [d]									
Normal weight	0.022	0.017	0.021	0.035*	0.021	0.013	0.010	0.019	0.020
Overweight	0.025	0.018	0.021*	0.038*	0.024	0.014	0.011	0.022	0.014
Obese class I	0.020	0.017	0.018	0.033*	0.019	0.013	0.005	0.017	0.018
Obese class II	0.009	0.006	0.006	0.022	0.010	0.005	-0.005	0.010	0.008
Obese class III	-0.004	-0.011	-0.010	0.044	-0.010	-0.008	-0.026	-0.009	-0.018
Laterality [e]									
Left	-0.007*	-0.002	-0.002	-0.002	-0.002	-0.002	-0.002	-0.002	-0.002
Charnley class [f]									
Category B	-0.070**	-0.063***	-0.087***	-0.150***	-0.116***	-0.040***	-0.075***	-0.091***	-0.110***
Category C	-0.185***	-0.168***	-0.196***	-0.291***	-0.231***	-0.115***	-0.182***	-0.196***	-0.241***
Hip pain level [g]									
Very mild	-0.077***	-0.057***	-0.077***	-0.119***	-0.087***	-0.037***	-0.050***	-0.067***	-0.080***
Mild	-0.138***	-0.108***	-0.121***	-0.154***	-0.123***	-0.074***	-0.094***	-0.105***	-0.132***
Moderate	-0.265***	-0.246***	-0.248***	-0.262***	-0.309***	-0.175***	-0.362***	-0.330***	-0.439***
Severe	-0.325***	-0.340***	-0.329***	-0.339***	-0.432***	-0.258***	-0.548***	-0.550***	-0.669***
Adjusted R^2	0.388	0.498	0.546	0.533	0.491	0.475	0.407	0.449	0.437
MAE	0.131	0.087	0.091	0.140	0.120	0.061	0.099	0.107	0.134
Normalized MAE	0.134	0.121	0.129	0.120	0.112	0.097	0.082	0.066	0.084
RMSE	0.169	0.117	0.119	0.174	0.158	0.085	0.160	0.153	0.196
Normalized RMSE	0.173	0.164	0.168	0.149	0.147	0.136	0.133	0.094	0.123
Spearman Rank correlation	0.631	0.717	0.736	0.730	0.727	0.724	0.724	0.711	0.715

Reference group: [a] Class I; [b] < 50; [c] Male; [d] Underweight; [e]Right; [f]Category A; [g] None; [h] hypothetical perspective; *P-value<0.05; **P-value<0.01; ***P-value<0.001

Table S11: Post hoc Bonferroni adjusted p-values of pairwise tests of the one-way ANOVA test for abilities of value sets to differentiate among ASA classes 6 years postoperatively (n=21,305)

Comparison	P-value							
	Swedish VAS	German VAS	Danish VAS[a]	UK VAS[a]	Swedish TTO	German TTO[a]	Danish TTO[a]	UK TTO[a]
Class I, Class II	<0.001	<0.001	<0.001	<0.001	<0.001	<0.001	<0.001	<0.001
Class I, Class III	<0.001	<0.001	<0.001	<0.001	<0.001	<0.001	<0.001	<0.001
Class I, Class IV	<0.001	<0.001	<0.001	<0.001	<0.001	0.001	0.001	<0.001
Class II, Class III	<0.001	<0.001	<0.001	<0.001	<0.001	<0.001	<0.001	<0.001
Class II, Class IV	0.017	0.095	0.094	0.066	0.006	0.070	0.133	0.062
Class III, Class IV	1.000	1.000	1.000	1.000	1.000	1.000	1.000	1.000

[a] Hypothetical perspective

Table S12: Kruskal Wallis rank sum Test to assess abilities of value sets to differentiate HRQoL by ASA class, 6 years postoperatively (n=21,305)

ASA class	Kruskal Wallis X^2							
	Swedish VAS	German VAS	Danish VAS	UK VAS	Swedish TTO	German TTO	Danish TTO	UK TTO
Class I<br>Class II<br>Class III<br>Class IV	1146.2*	1082.1*	1196.2*	1105.2*	1187.7*	1019.2*	1083.5*	1046.1*

[a]hypothetical perspective
*P-value<0.001

A list of R packages used in the analysis

1. **car: Companion to Applied Regression**

   Fox J, Weisberg S (2019). *An R Companion to Applied Regression*, Third edition. Sage, Thousand Oaks CA. https://socialsciences.mcmaster.ca/jfox/Books/Companion/.

2. **dplyr**

   Hadley Wickham, Romain François, Lionel Henry and Kirill Müller (2019). dplyr: A Grammar of Data Manipulation. R package version 0.8.3. https://CRAN.R-project.org/package=dplyr

3. **ggplot2**

   H. Wickham. ggplot2: Elegant Graphics for Data Analysis. Springer-Verlag New York, 2016.

4. **gvlma**

   Edsel A. Pena and Elizabeth H. Slate (2019). gvlma: Global Validation of Linear Models Assumptions. R package version 1.0.0.3. https://CRAN.R-project.org/package=gvlma

5. **lmtest**

   Achim Zeileis, Torsten Hothorn (2002). Diagnostic Checking in Regression Relationships. R News 2(3), 7-10. URL https://CRAN.R-project.org/doc/Rnews/

6. **Metrics**

   Ben Hamner and Michael Frasco (2018). Metrics: Evaluation Metrics for Machine Learning.R package version 0.1.4. https://CRAN.R-project.org/package=Metrics

7. **sandwich**

   Zeileis A (2004). "Econometric Computing with HC and HAC Covariance Matrix Estimators."_Journal of Statistical Software_, *11*(10), 1-17. doi: 10.18637/jss.v011.i10 (URL:https://doi.org/10.18637/jss.v011.i1).

8. **SimDesign**

   Phil Chalmers (2020). SimDesign: Structure for Organizing Monte Carlo Simulation Designs. R package version 2.0.1. https://CRAN.R-project.org/package=SimDesign

9. **sjstats**

   Lüdecke D (2019). _sjstats: Statistical Functions for Regression Models (Version 0.17.7). doi: 10.5281/zenodo.1284472 (URL: https://doi.org/10.5281/zenodo.1284472),<URL: https://CRAN.R-project.org/package=sjstats>.

10. **RVAideMemoire**

    Maxime Hervé (2020). RVAideMemoire: Testing and Plotting Procedures for Biostatistics.R package version 0.9-74. https://CRAN.R-project.org/package=RVAideMemoire

11. **DescTools**

    al. ASem (2020). DescTools: Tools for Descriptive Statistics. R package version 0.99.34, https://cran.r-project.org/package=DescTools.

12. **Kendall**

    A.I. McLeod (2011). Kendall: Kendall rank correlation and Mann-Kendall trend test http://www.stats.uwo.ca/faculty/aim
